# Proteomics Study Reveals the Anti-Depressive Mechanisms and the Compatibility Advantage of Chaihu-Shugan-San in a Rat Model of Chronic Unpredictable Mild Stress

**DOI:** 10.3389/fphar.2021.791097

**Published:** 2022-01-17

**Authors:** Xiaofei Zhu, Teng Li, En Hu, Lihua Duan, Chunhu Zhang, Yang Wang, Tao Tang, Zhaoyu Yang, Rong Fan

**Affiliations:** ^1^ Department of Integrated Traditional Chinese and Western Medicine, Institute of Integrative Medicine, Xiangya Hospital, Central, South University, Changsha, China; ^2^ National Clinical Research Center for Geriatric Disorders, Xiangya Hospital, Central South University, Changsha, China

**Keywords:** traditional Chinese medicine, Chaihu-Shugan-San, overall effects, decomposed recipes, proteomics, depression

## Abstract

**Background:** Chaihu-Shugan-San is a classical prescription to treat depression. According to the traditional Chinese medicine (TCM) principle, the 2 decomposed recipes in Chaihu-Shugan-San exert synergistic effects, including Shu Gan (stagnated Gan-Qi dispersion) and Rou Gan (Gan nourishment to alleviate pain). However, the specific mechanism of Chaihu-Shugan-San on depression and its compatibility rule remain to be explored.

**Objective:** We aimed to explore the anti-depression mechanisms and analyze the advantage of TCM compatibility of Chaihu-Shugan-San.

**Methods:** The chronic unpredictable mild stress (CUMS) rat model was established. Antidepressant effects were evaluated by sucrose preference test (SPT), and forced swimming test (FST). Tandem Mass Tag (TMT)-based quantitative proteomics of the hippocampus was used to obtain differentially expressed proteins (DEPs). Bioinformatics analysis including Gene Ontology (GO), pathway enrichment, and protein-protein interaction (PPI) networks was utilized to study the DEPs connections. At last, the achieved key targets were verified by western blotting.

**Results:** Chaihu-Shugan-San increased weight gain and food intake, as well as exhibited better therapeutic effects including enhanced sucrose preference and extended immobility time when compared with its decomposed recipes. Proteomics showed Chaihu-Shugan-San, Shu Gan, and Rou Gan regulated 110, 12, and 407 DEPs, respectively. Compared with Shu Gan or Rou Gan alone, the expression of 22 proteins was additionally changed by Chaihu-Shugan-San treatment, whereas the expression of 323 proteins whose expression was changed by Shu Gan or Rou Gan alone were not changed by Chaihu-Shugan-San treatment. Bioinformatics analysis demonstrated that Chaihu-Shugan-San affected neurotransmitter’s release and transmission cycle (e.g., γ-aminobutyric acid (GABA), glutamate, serotonin, norepinephrine, dopamine, and acetylcholine). GABA release pathway is also targeted by the 22 DEPs. Unexpectedly, only 2 pathways were enriched by the 323 DEPs: Metabolism and Cellular responses to external stimuli. Lastly, the expression of Gad2, Vamp2, and Pde2a was verified by western blotting.

**Conclusions:** Chaihu-Shugan-San treats depression *via* multiple targets and pathways, which may include regulations of 110 DEPs and some neurotransmitter’s transmission cycle. Compared with Shu Gan and Rou Gan, the 22 Chaihu-Shugan-San advanced proteins and the affected GABA pathway may be the advantages of Chaihu-Shugan-San compatibility. This research offers data and theory support for the clinical application of Chaihu-Shugan-San.

## Introduction

Depression is one of the most pervasive, disabling, and expensive of all neuropsychiatric disorders (Porcu et al., 2020). According to the report of WHO, depression will rank as the first reason for global disease burden in 2030 (Malhi and Mann, 2018). Despite large efforts are put into developing therapies for depression, western medications are still invalid in myriad cases and regularly cause insufferable side effects (Duman and Aghajanian, 2012).

Therefore, the formulas of Traditional Chinese medicine (TCM), as an essential alternative and complementary medicine, are gaining more and more enthusiasts among the patients (Wang et al., 2017). The formulas of TCM have been widely utilized to recover the harmony disturbed in diseases through multi-target synergistic functions of its corresponding components, hence qualified and efficacious for the treatment of complicated diseases such as depression (Yeung et al., 2014). Considerably, increasing clinical evidence demonstrates the desirable efficiency of the prescription of TCM in treating depression (Peng et al., 2015; Chi et al., 2019; Wang et al., 2019). Since ancient times, Chaihu-Shugan-San, a classic TCM prescription was written in *Jing Yue Quan Shu*, has been extensively utilized in Chinese clinical applications for the treatment of depression (Liu et al., 2020). It consists of 7 crude herbs, namely Chai Hu (*Bupleurum chinense DC.*), Chen Pi (*Citrus reticulata Blanco*), Xiang Fu (*Cyperus rotundus L.*), Zhi Qiao (*Citrus × aurantium L.*), Chuan Xiong (*Ligusticum striatum DC.*), Bai Shao (*Paeonia lactiflora Pall.*), and Gan Cao (*Glycyrrhiza uralensis Fisch.*) in a specific ratio of 4: 4: 3: 3: 3: 3: 1 (Table 1). All plant names or species were validated using http://www.theplantlist.org/. Modern studies show that Chaihu-Shugan-San has significant clinical efficacy in depression (Kim et al., 2005; Li et al., 2010; Wang et al., 2012; Qiu et al., 2014a; Qiu et al., 2014b). Our previous studies suggest that regional cerebral blood flow perfusion defects and clinical symptoms of depression can be improved by Chaihu-Shugan-San, the mechanisms involve reversing the hypothalamic-pituitary-adrenal (HPA) axis hyperfunction (Qiu et al., 2014a). Other researches support that Chaihu-Shugan-San exerts antidepressant effect by increasing monoamine neurotransmitters, regulating brain-derived neurotrophic factor (BDNF), and affecting the BDNF-TrkB-ERK/Akt signalling pathway (Li et al., 2009; Qiu et al., 2014b; Liu Q. et al., 2018; Chen et al., 2018). Despite these researches, the mechanisms of the multi-component Chaihu-Shugan-San are far from being fully understood, which limits its application.

**TABLE 1:** Composition and corresponding ratio of Chaihu-Shugan-San, Shu Gan, and Rou Gan.

Group	Herb	Chinese name	Latin name	Ratio	Medicinal part	Batch number
Shu Gan	*Bupleurum chinense DC.*	Chai Hu	*Radix Bupleuri*	4	Root	20051802
Shu Gan	*Citrus reticulata Blanco*	Chen Pi	*Pericarpium Citri Reticulatae*	4	Pericarp	20071804
Shu Gan	*Cyperus rotundus L.*	Xiang Fu	*Rhizoma Cyperi*	3	Root	20060202
Shu Gan	*Citrus × aurantium L.*	Zhi Qiao	*Fructus Aurantii*	3	Fruit	20072111
Rou Gan	*Ligusticum striatum DC.*	Chuan Xiong	*Rhizoma Chuanxiong*	3	Rhizome	20042708
Rou Gan	*Paeonia lactiflora Pall.*	Bai Shao	*Radix Paeoniae Alba*	3	Root	20041711
Rou Gan	*Glycyrrhiza uralensis Fisch.*	Gan Cao	*Radix Glycyrrhizae*	1	Root and rhizome	20040108

From the perspective of TCM theory, a formula is composed of several compatibility groups that exert different but synergistic functions (Zhang et al., 2015). Clinically, strive to achieve the best therapeutic effect and minimal side effects, TCM doctors would combine traditional Chinese medicinal material groups according to TCM theory. Through organic integration, the TCM prescription has applicability to deal with the disease complexity. In TCM theory, depression is considered to be Gan-Qi stagnation and further would be upgraded as Gan malnutrition (Scheid, 2013). Chaihu-Shugan-San can disperse stagnated Gan-Qi and nourish Gan to alleviate pain because consists of 2 recipes: Shu Gan, which disperses stagnated Gan-Qi; and Rou Gan, which nourishes Gan to alleviate pain (Table 1) (Qiu et al., 2014b). However, the mechanisms of the TCM theory-based synthetic effects of Rou Gan and Shu Gan subdivisions in Chaihu-Shugan-San are not reported.

Chaihu-Shugan-San improves depression outcomes via multi-targets and multi-pathways (Li et al., 2009; Qiu et al., 2014b; Li et al., 2014). Considering the complex changes in protein expression *in vivo* during depressive states, proteomics is considerably potent for monitoring the dynamic alternations (Zhang J. et al., 2016). The technique is a method for accurately detecting the changes of protein in disease states and after treatment by drugs (Zhang L. et al., 2016). Previous proteomic researches of postmortem depression cases and rodent depression models have shown dysfunctions of energy metabolism, synaptic plasticity, neurogenesis, and neurotransmission (Zhang et al., 2018). Collectively, proteomics provides holistic insights into the disease- and drug-driven pathway alteration, which in turn gains evidence to identify treatment targets. Thus, this strategy is hopeful to better understand the multiple mechanisms of Chaihu-Shugan-San and its decomposed recipes in depression treatment, which has not been published before.

In the current investigation, Tandem Mass Tag (TMT) labeling quantitative proteomics was carried out to explore the hippocampus proteins in the rats having chronic unpredictable mild stress (CUMS) and to analyzed the differentially expressed proteins (DEPs). With the bioinformatics method, we next elucidated the underlying mechanisms of Chaihu-Shugan-San in treating depression and compared the effects of Chaihu-Shugan-San with those of its decomposed recipes (Shu Gan and Rou Gan). Our study will highlight the synthetical anti-depressive superiority of the Chaihu-Shugan-San formula.

## Materials and Methods

### Preparation of Chaihu-Shugan-San, Shu Gan, and Rou Gan

The composition and corresponding ratio of Chaihu-Shugan-San and its decomposed recipes (Shu Gan and Rou Gan) are listed in Table 1. The herbs were acquired from Hunan Zhenxing Chinese Medicine Co., Ltd (Hunan, China. Drug Manufacturing Certificate: NO.20150021, Drug GMP certificate: HN20150147). The authentication of the purchased herbs was performed via Professor Peng Lei, Department of Chinese herbal medicine of Central South University (CSU, Changsha, China). At Xiangya Hospital of CSU, their voucher samples were deposited. The herbs were soaked in 25°C water for 0.5 h, then heated to 100°C and boiled for 30 min. The first filtrate was collected in a beaker. The medicine herb residue in the same volume of water was refluxed and for 30 min was heated, then the second filtrate was collected. The above 2 filtrates were integrated and filtered by the 5-layered cotton gauze. The concentrated solution was stored at 4°C (Yan et al., 2020).

### Detection of the Chaihu-Shugan-San Components Utilizing Ultra-Performance Liquid Chromatography

The measurement of UPLC chromatographic was conducted while employing a Waters ACQUITY UPLC series supplied with a quaternary pump, a diode array detector (PDA), an online degasser, and an autosampler controlled by Empower2. For chromatographic separation, Waters BEH C18 column (2.1 × 50 mm, 1.7 μm) was utilized. The mobile phase included acetonitrile (A) and acetic acid (pH = 3.5) (B). The curves of gradient elution were: 0–10 min, 5%A: 95%B; 10–20 min, 15%A: 85%B; 20–30 min, 30%A: 70%B; 30–40 min, 50%A: 50%B; 40–45 min, 70%A: 30%B; 45–50 min, 80%A: 20%B. The PDA was set at 190–480 nm. The parameters were set as follows: Column temperature: 40°C; Injection volume: 3 ul; Flow rate: 0.5 ml/ min.

### Animal Experimental Design

Male Sprague-Dawley (SD) Specific pathogen-free (SPF) rats (180–220 g) were procured from the Experimental Animals Centre of CSU, Changsha, Hunan. The rats were accommodated 3 per cage and received water and food ad libitum in circumstances of the normal light-dark cycle, room relative humidity (40–60%), room noise (30–50 dB), and room temperature (19–25°C). The rats adapted for 7 days and were divided into 6 groups randomly: Control, Model, Fluoxetine, Chaihu-Shugan-San, Shu Gan, and Rou Gan (*n* = 8 per group).

After the 7 days habituation period, except for rats in the control group, the procedure of CUMS was performed on all of the animals (Antoniuk et al., 2019). The procedure of CUMS was already designed and explained with slight changes (Xing et al., 2019). The CUMS-induced rats were held in individual cages for 4 weeks and subjected to different stressors (a random stressor per day): no water (1 d), no food (1 d), wet cage (1 d), hot swimming (5 min, 45°C), tail pinch (5 min), restraint stress (3 h), cold swimming (5 min, 4°C), cage tilting (1 d), light or dark (1 d), noise stimulus (2 h). To ensure unpredictability of stressors, the rats did not face a similar stressor on 2 consecutive days.

**FIGURE 1:**
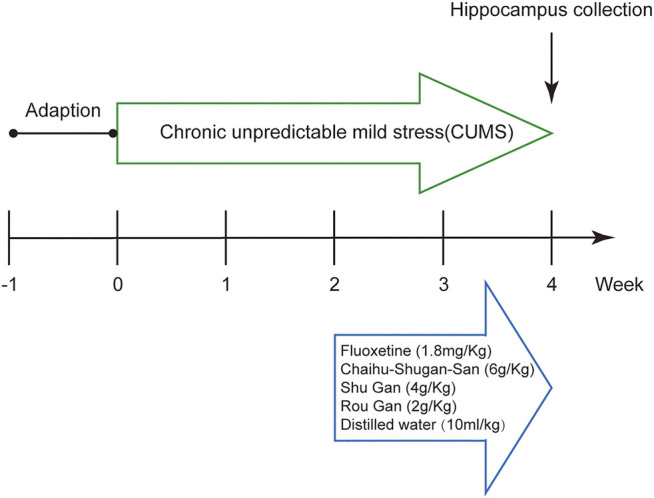
Schematic representation of the experimental procedure.

All drugs were intragastrically administered daily after 2 weeks CUMS as follows: Chaihu-Shugan-San 6 g/ kg; Shu Gan 4 g/kg; Rou Gan 2 g/kg; Fluoxetine 1.8 mg/ kg; distilled water 10 ml/ kg for 2 weeks. The capsules of fluoxetine hydrochloride were bought from Lilly Suzhou Pharmaceutical Co., Ltd (20 mg/granules, Batch number: J20170022). The dosage of the drugs administered to the rats were converted from that of a 70 kg-adult (All procedures were shown in Figure 1).

Body weighing, food intake measurement, forced swimming test (FST), and sucrose preference test (SPT) on each rat were conducted on day 1, 14, and 28 of the experiment, respectively (Xing et al., 2019). On day 28, after the SPT, the rats were anesthetized intraperitoneally with 3% pentobarbital sodium (65 mg/ kg) and then perfused transcardially with 0.9% sterile saline (250 ml, 4°C). Hippocampus is the key brain region for stress response and emotion regulation (Snyder et al., 2011). Therefore, hippocampi were rapidly taken out and stored at a −80°C refrigerator.

Our experiments were performed conform to the National Guidelines for Experimental Animal Welfare (MOST, PR China, 2006), which possessed complete approval from the Association for Assessment and Accreditation of Laboratory Animal Care International (AAALAC Intl.). The experimental processes were complied with the guidelines of laboratory animal care and confirmed through the Medical Ethics Committee of CSU (Approval number: 2020sydw0892).

### Weight Gain and Food Intake

The weight and food consumption of each animal were assessed, then the values from the same time (Day 1, 14, and 28) were compared among the 6 groups. Weight change (%)= (weight-weight on day 1)/weight on day 1 × 100% (Li et al., 2005).

### Behavioral Tests

Behavioral alterations were assessed by the FST and the SPT, which are classical behavioral tests that measure core symptoms of depression and have been extensively executed to evaluate the efficacy of the antidepressant drug (Xing et al., 2015; Hu et al., 2017; Xing et al., 2019).

#### SPT

The test was carried out as already explained with a slight change (Xing et al., 2019; Yan et al., 2020). The trial was initiated by training the rats for adapting to 1% sucrose solution (w/v). Following the adaptation, rats were not allowed access to water and food for 1 day. Rats were presented with 2 bottles: the 2 bottles were filled with 200 ml water and 1% solution of sucrose, respectively. Sucrose preference (%) = sucrose consumption/(sucrose consumption + water consumption) × 100%. Water and sucrose solution consumption in 1 h were assessed via subtracting the weight of the bottles.

#### FST

As described previously, in the pretest, the animals were transferred into a plexiglass cylinder (25 cm in diameter, 45 cm deep) with a depth of 35 cm water for 15 min (23–25°C) (Xing et al., 2019). Approximately 1 d after the pretest, the rats were reintroduced into the same cylinder separately. The rats were forced to swim for 6 min and in the last 4 min, the time of immobility was recorded. Following each session of swimming, by using a clean towel, the rats were thoroughly dried, and after 20 min of resting were returned to their home cage.

### TMT Labeled Quantitative Proteomics

**FIGURE 2:**
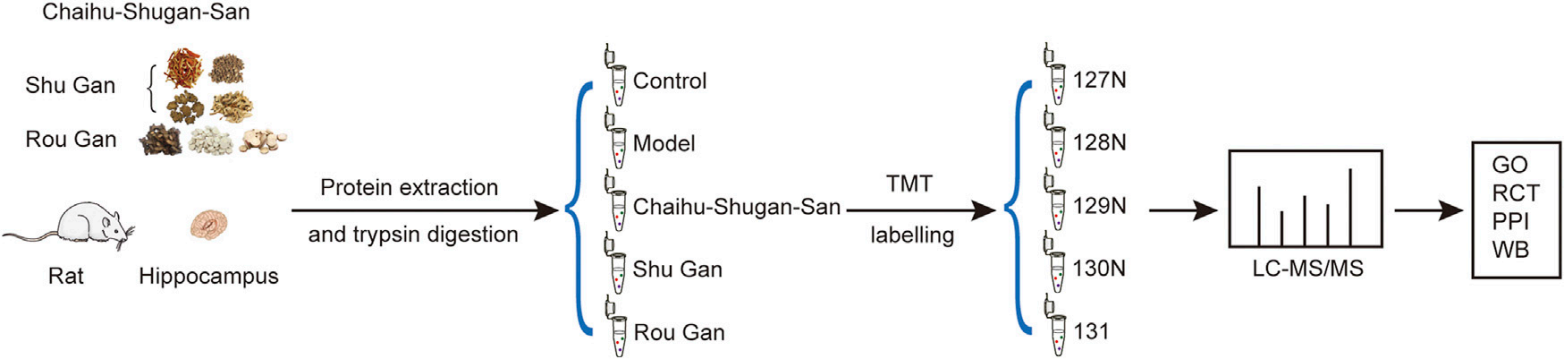
Schematic representation of the hippocampus protein profiling. (GO: Gene Ontology. RCT: Reactome pathway. PPI: Protein-Protein Interaction. WB: Western blotting).

As described in Figure 2, hippocampi were removed for TMT labeling and protein extraction after the 2-weeks gavage period. Then, LC-MS/MS were utilized to acquire raw data and carried out the analysis.

#### Protein Sample Preparation and TMT Tag Labeling

Hippocampus samples were homogenized in the working liquid (the RIPA lysate mixed with a protease inhibitor). Next, the centrifuge of the samples was done for 15 min at 14000 g (4°C). The supernatants were transferred to the tubes of EP and the concentration of protein was calculated via the kit of BCA Quantification (Thermo Scientific, United States). The 100 μg proteins per sample were subjected to reduction, alkylation, acetone precipitation, and re-solution. The achieved peptides attained from each group were tagged with TMT-127N, TMT-128N, TMT-129N, TMT-130N, and TMT-131 respectively. TMT labeling was carried out conforming to the protocol of the producer (Thermo Scientific, United States).

#### Reversed-phase High pH Fractionation

Desalination of the peptides with TMT labels obtained from five groups was carried out using a C_18_ column (Dr. Maisch, Germany). Next, the peptides were gathered and dried by vacuum. The 100 μg peptide samples were separated by reversed-phase HPLC (Thermo Scientific, United States) at pH = 10. Chromatographic column: 150 mm × 2.1 mm (waters, XBridge BEH C 18 XP Column); Mobile phases A: 10 mM ammonium formate aqueous solution, pH = 10; Mobile phases B: 10 mM ammonium formate, 90% ACN, 10% H_2_O, pH = 10. Liquid phase gradient 120 min, mobile phase B: 5% for 2 min, 5–28% for 78 min, 28–50% for 12 min, 50–80% for 2 min, 80% for 4 min, 80–5% for 2 min and 5% for 20 min. One fraction was collected in 40 s intervals. A total of 180 fractions were collected and combined to obtain 20 fractions. The fractions were subjected to dried by vacuum and followed by storage at −80°C.

#### LC-MS/MS Analysis

For each group, 1 μg of peptides were divided via the system of nano-UPLC liquid phase system (EASY-nLC1200) and the detection was executed via the mass spectrometer of Q-Exactive (Thermo Finnigan) with 2 technical repeats per component. The separation of peptides mixture was performed by the 100 μm ID × 15 cm reversed-phase column (Reprosil-Pur 120 C18-AQ, 1.9 μm, Dr. Math). The column was equilibrated with 100% solvent A (0.1% formic acid in acetonitrile aqueous solution, contain 2% acetonitrile). The sample was separated by the column at 300 nL/ min flow rate and 90 min linear gradient. Mobile phase B (0.1% formic acid in acetonitrile aqueous solution, contain 80% acetonitrile): 6–28% for 70 min, 28–40% for 12 min, 40–100% for 2 min, 100% for 2 min, 100–2% for 2 min, and 2% for 2 min. Then, by the mass spectrometer of Q-Exactive (90 min/sample), the separated peptides were assessed.

#### Proteins Identification and Quantitation

Raw data of LC-MS/MS were quantitatively studied and explored via MaxQuant (Version 1.5.6.0) and the Uniprot database. The criteria were as follows (not described value were default): the quantitation type was TMT 3-plex; sites of labeling were peptide N-terminal and Lys (K) (PIF = 0.75); the maximum number of missed cuts was 2; the specific enzyme was trypsin/P; Variable modification was Acetyl (protein N-term) and Oxidation (M); the maximum peptide molecular weight was 4600 Da and minimum peptide length was 7; fixed modification was Carbamidomethyl (C); only quantified unmodified unique peptide and FDR < = 0.01 (Wilhelm et al., 2014). Subsequently, the quantitative proteomics results were analyzed to obtain the DEPs through the criteria as given below: FDR<=0.01, unique peptides > = 2, average ratio-fold change (FC) > 1.2 (up-regulation) or <0.83 (down-regulation), and *p*-value < 0.05 (Ren et al., 2013; Zhou et al., 2020).

### Bioinformatics Analysis

GO annotation (biological process, molecular function, and cellular component), enrichment of pathway, and the networks of protein-protein interaction (PPI) were utilized to analyze the connections of the DEPs. The DAVID (https://david.ncifcrf.gov/, RRID: SCR_001881) database was utilized to obtain GO annotation. The Reactome Pathway (https://reactome.org/, RRID: SCR_003485) database and The STRING (https://string-db.org/, RRID: SCR_005223) database were used to enrich pathways and get PPI networks (Haw and Stein, 2012; Jassal et al., 2020).

### Western Blotting

By using a RIPA lysis buffer (100 ul/0.01 g), hippocampi were homogenized and subsequently were centrifuged for 10 min at 12000 rpm (4°C). The concentrations of protein were assayed with the method of BCA. The separation of the proteins was done on 12% polyacrylamide SDS-PAGE gel and afterward were transferred to the membranes of PVDF. The membranes were incubated with primary antibodies as follows: rabbit anti-Gad2 (1:5000, Proteintech, Cat# 20746-1-AP, RRID: AB_10949196), rabbit anti-Vamp2 (1:2000, Proteintech, Cat# 10135-1-AP, RRID: AB_2256918), rabbit anti-Pde2a (1:500, Proteintech, Cat# 55306-1-AP, RRID: AB_11182279) and mouse anti-β-actin (1:5000, Proteintech, Cat# 66009-1-Ig, RRID: AB_2687938) overnight at 4°C. After being washed by PBST, the membranes were incubated with secondary antibodies as follows: HRP goat anti-mouse IgG (1:5000, Proteintech, Cat# SA00001-1, RRID: AB_2722565) or HRP goat anti-rabbit IgG (1:6000, Proteintech, Cat# SA00001-2, RRID: AB_2722564) for 90 min at 25°C. The incubation of the membranes was carried out for 5min with the solution of ECL chemiluminescent solution (Thermo Scientific, United States) and wrapped in the plastic sealing film. Subsequently, the membranes were subjected to a film of X-ray in the dark box for 5 s-20 min. The bands of protein were quantified and visualized utilizing ImageJ (RRID: SCR_003070) computer program.

### Statistic Analysis

The difference among groups was calculated by one-way analysis of variance (ANOVA) followed by Tukey HSD. *p*-value < 0.05 was set as the significant threshold. Data were analyzed by SPSS 22.0 software (International Business Machines Corp, Armonk, NY, United States, RRID: SCR_019096). The achieved data were expressed as the mean ± SEM.

## Results

### UPLC Analysis the Components of Chaihu-Shugan-San

To ensure the quality of the herbal within the Chaihu-Shugan-San, UPLC was carried out with acetic acid and acetonitrile for chromatogram separation. Using the separation conditions described in Materials and methods, 9 compounds from Chaihu-Shugan-San, including Albiflorin std, Ferulic acid, Paeoniflorin, Naringin, Hesperidin, Hydrated hesperidin, Neohesperidin, Ammonium glycyrrhizinate, and Glycyrrhetinic acid were identified (Supplementary Figure S1). A total of 9 characteristic peaks were well resolved.

### The Synthetical Antidepressant Effects of Chaihu-Shugan-San

#### Chaihu-Shugan-San Increased Weight Gain and Food Intake in the CUMS-Induced Rats

**FIGURE 3:**
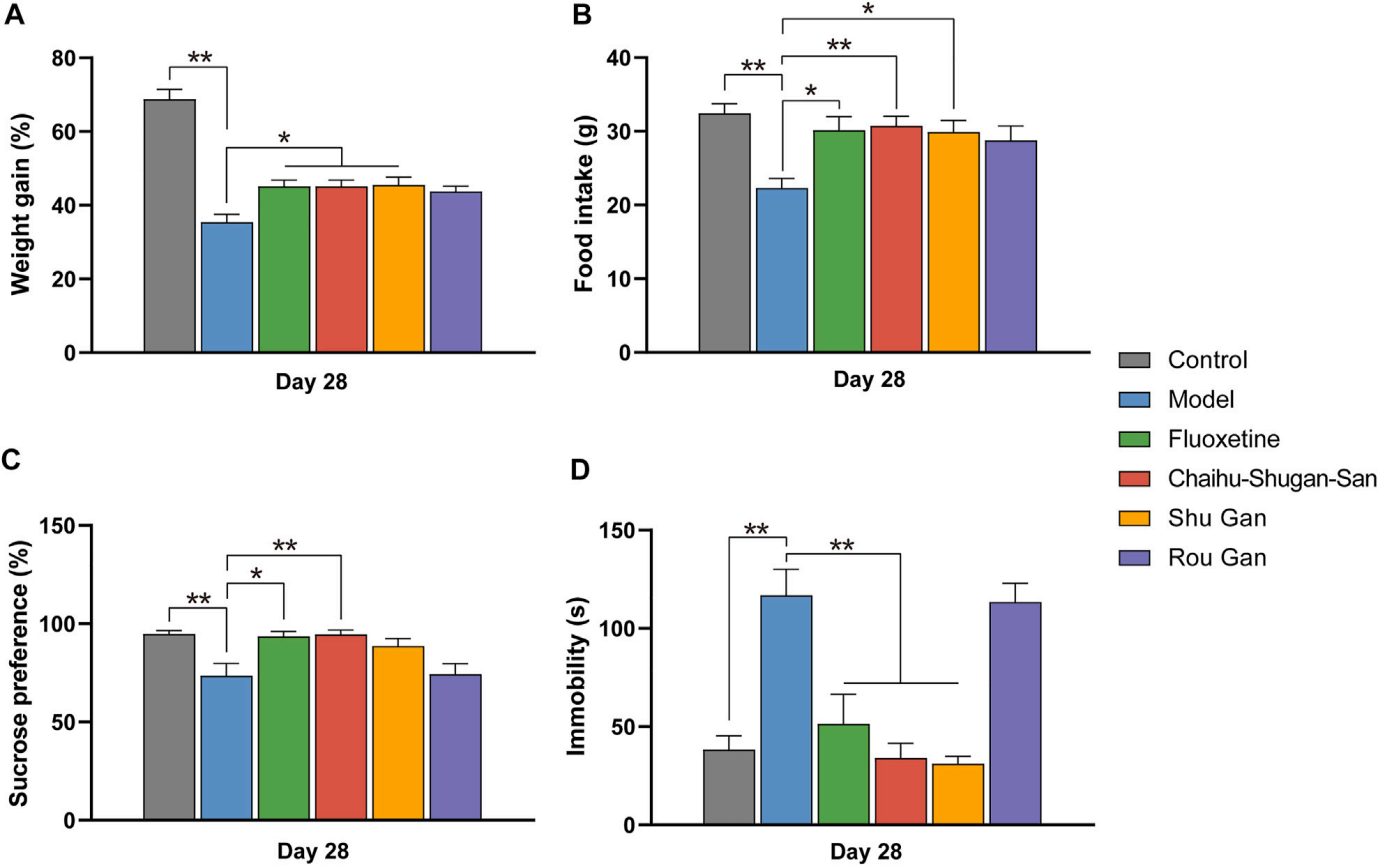
The therapeutic effects of Fluoxetine, Chaihu-Shugan-San, Shu Gan, and Rou Gan in treating CUMS rats. (*n* = 8, ∗*p* < 0.05, ∗∗*p* < 0.01, data are presented as mean ± SEM). Bodyweight gain **(A)**, Food intake **(B)**, sucrose consumption test **(C)**, and forced swim test **(D)** were performed on day 28.

On day 1, 14, and 28, the body weights of the animals were observed to calculate the rate of weight gain. At the same time, food intake was also recorded. We observed no significant difference in baseline body weights and food intake between the 6 groups (Supplementary Figure S2A). Notably, the weight gain and food intake of the CUMS group was lesser in comparison with the control group on day 14 but didn’t reach the level of statistical significance (Supplementary Figures S3A,B). After treatment, the weight gain (F_5,42_ = 31.792, *p* < 0.01) and food intake (F_5,42_ = 5.033, *p* < 0.01) of the Chaihu-Shugan-San group, the fluoxetine group, and the Shu Gan group was significantly higher in comparison with the model group (*p* < 0.05) (Figures 3A,B).

#### Chaihu-Shugan-San Exerted an Antidepressant-like Effect on the CUMS-Induced Rats

SPT was implemented on day 1, 14, and 28. There existed no significant discrepancies between the various groups before CUMS (Supplementary Figure S2B). On day 14 (F_5,42_ = 4.123, *p* < 0.01), the sucrose preference index was significantly reduced within the groups of CUMS in comparison with the control group (*p* < 0.05) (Supplementary Figure S3C). After 2 weeks of treatments (F_5,42_ = 6.211, *p* < 0.01), the sucrose preference index of the fluoxetine group (*p* < 0.05) and the Chaihu-Shugan-San group (*p* < 0.01) was significantly increased than the model group, suggesting that Chaihu-Shugan-San and fluoxetine can improve the lack of pleasure created by CUMS. The sucrose preference index of the Shu Gan group and the Rou Gan group was no statistical significance in comparison with the model group (Figure 3C).

The FST was measured on day 1, 14, and 28. There were none of the behaviors were significantly different among the groups on day 1 (Supplementary Figure S2C). On day 14 (F_5,17.854_ = 62.339, *p* < 0.01), the immobility time was significantly increased within the groups of CUMS in comparison with the control group (*p* < 0.01) (Supplementary Figure S3D). Furthermore, after 2 weeks of treatment (F_5,42_ = 15.607, *p* < 0.01), the immobility time of the fluoxetine group, the Chaihu-Shugan-San group, and the Shu Gan group were significantly lower than the model group (*p* < 0.01). But compared with the model group, the immobility time of the Rou Gan group had no statistical significance (Figure 3D).

### Proteomics Analysis of DEPs

**FIGURE 4:**
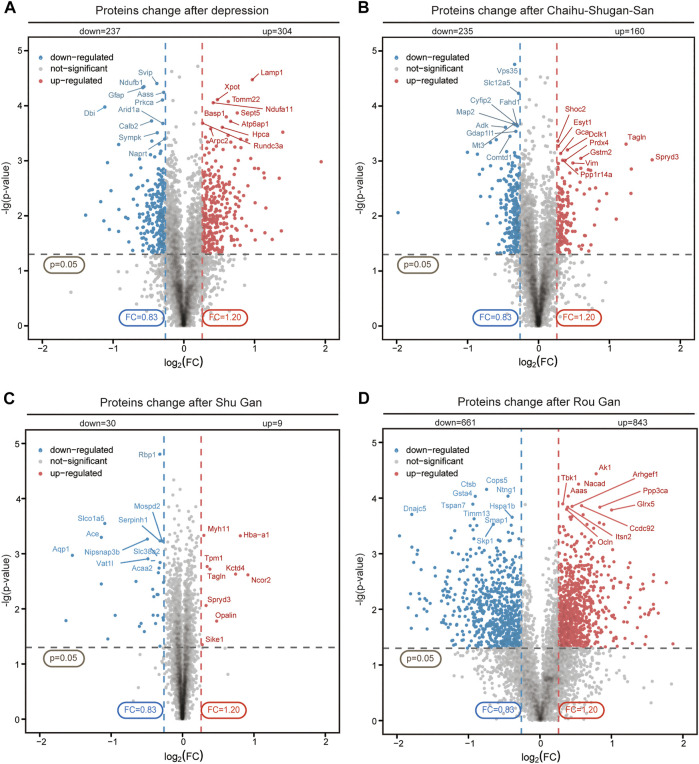
Quantitative analysis and identification of proteins. The up-regulated (red) or down-regulated (blue) proteins between Model *vs.* Control groups **(A)**, Chaihu-Shugan-San *vs.* Model groups **(B)**, Shu Gan *vs.* Model groups **(C)**, and Rou Gan *vs.* Model groups **(D)** of 5585 proteins were shown in the volcano plot.

TMT-based proteomics was used to obtain the DEPs of the hippocampus and to explore the potential antidepressant protein targets. A total of 73413 polypeptides and 7268 proteins were detected by TMT quantitative proteomics. We screened 5585 proteins with FDR<=0.01 and unique peptide matches > = 2. DEPs were screened out by *p*-value < 0.05 and FC (>1.2, up-regulated or <0.83, down-regulated). The volcano plot showed the relative changes in protein levels (Figure 4). In Model *vs.* Control group, 541 DEPs were identified (304 up-regulation and 237 down-regulation). In Chaihu-Shugan-San *vs.* Model group, 395 DEPs were identified (160 up-regulation and 235 down-regulation). In Shu Gan *vs.* Model group, 39 DEPs were identified (9 up-regulation and 30 down-regulation). In Rou Gan *vs.* Model group, 1504 DEPs were identified (843 up-regulation and 661 down-regulation).

**FIGURE 5:**
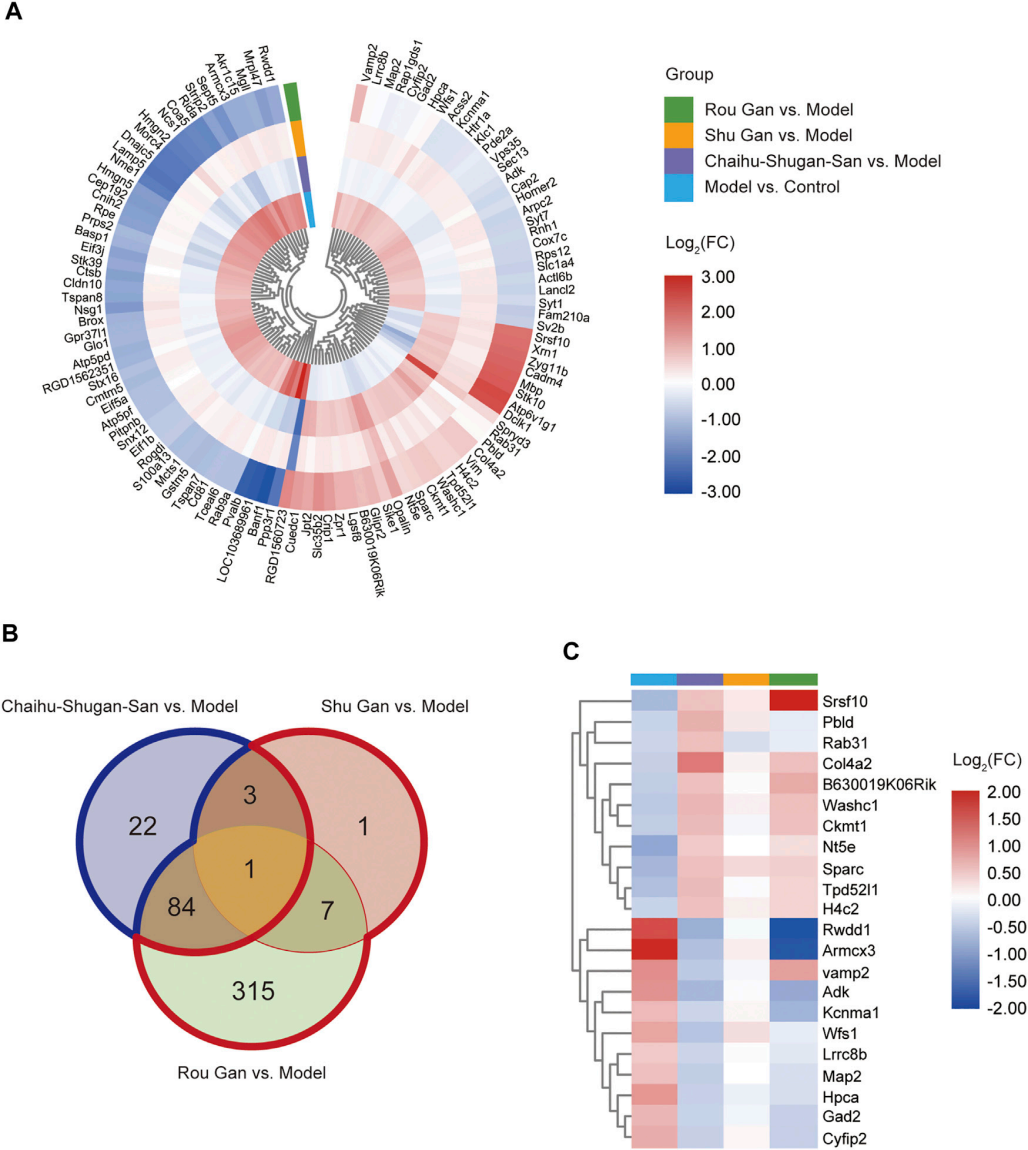
**(A)** Heat map analysis of 110 Chaihu-Shugan-San DEPs. **(B)** Venn diagrams of the DEPs associated with Chaihu-Shugan-San, Shu Gan, and Rou Gan in treating depression. (Blue border: the 22 Chaihu-Shugan-San advanced proteins; Red border: the 323 proteins whose expression was changed by Shu Gan or Rou Gan alone were not changed by Chaihu-Shugan-San treatment). **(C)** Heat map analysis of 22 Chaihu-Shugan-San advanced proteins. The color represents log_2_(FC) of DEPs, red indicates an increase and blue indicates a decrease. (FC: fold changes).

In both Model *vs.* Control group and Chaihu-Shugan-San *vs.* Model group, 126 overlapping DEPs were identified and 110 proteins were reversed by Chaihu-Shugan-San treatment among them. The elaborated information about 110 DEPs was showed in Figure 5A and listed in Supplementary Table S1. In both Model *vs.* Control group and Shu Gan *vs.* Model group, we identified the 14 overlapping DEPs. Among the 14 DEPs, 12 proteins were reversed by Shu Gan treatment (Supplementary Table S2). In both Model *vs.* Control group and Rou Gan *vs.* Model group, the 410 overlapping DEPs were identified. Among the 410 DEPs, 407 proteins were reversed by Rou Gan treatment (Supplementary Table S3). Among the reversed proteins (Figure 5B), 22 proteins whose expression was additionally changed by Chaihu-Shugan-San treatment compared with Shu Gan or Rou Gan alone were the advanced proteins of Chaihu-Shugan-San (Figure 5C). Whereas the expression of 323 proteins whose expression was changed by Shu Gan or Rou Gan alone were not changed by Chaihu-Shugan-San treatment.

### Bioinformatics Analysis of DEPs

#### Functional Classification of DEPs

**FIGURE 6:**
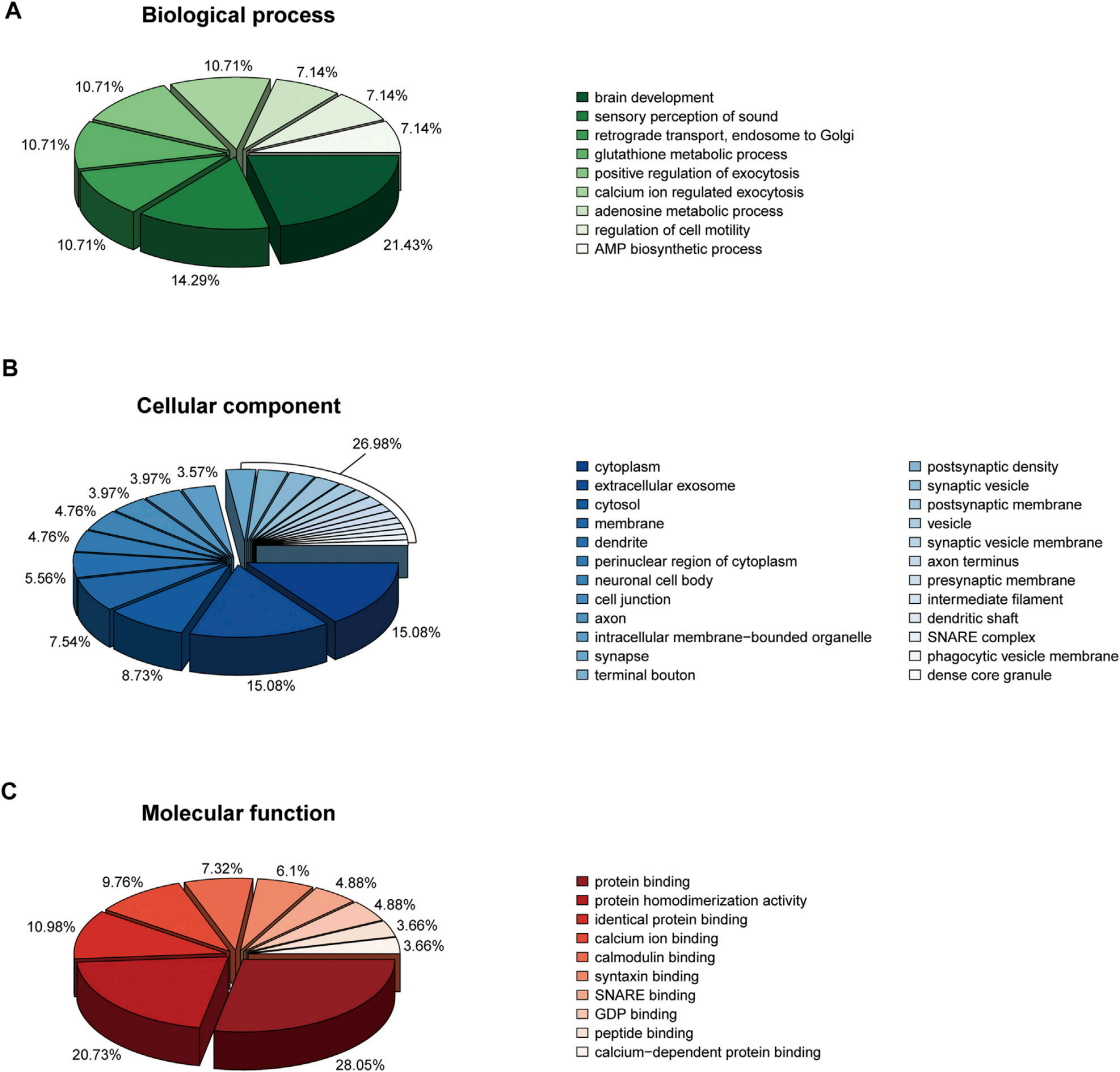
Bioinformatics analysis (GO annotation) of 110 DEPs. **(A)** Biological process. **(B)** Cellular component. **(C)** Molecular function.

When imported 110 DEPs to the DAVID database, we obtained 9, 24, 10 biological functions in biological process, cellular component, and molecular function, respectively (Figure 6). In the biological process, 110 DEPs enriched in brain development (*p* = 0.01591), glutathione metabolic process (*p* = 0.02633), and calcium ion regulated exocytosis (*p* = 0.00338) (Figure 6A). In the cellular component, 110 DEPs enriched in synapse (*p* = 0.00061), axon (*p* = 0.00007), and vesicle (*p* = 0.00819) (Figure 6B). In the molecular function, 110 DEPs enriched in protein binding (*p* = 1.11E-06), calcium ion binding (*p* = 0.03696), and SNARE binding (*p* = 0.00262) (Figure 6C).

**FIGURE 7:**
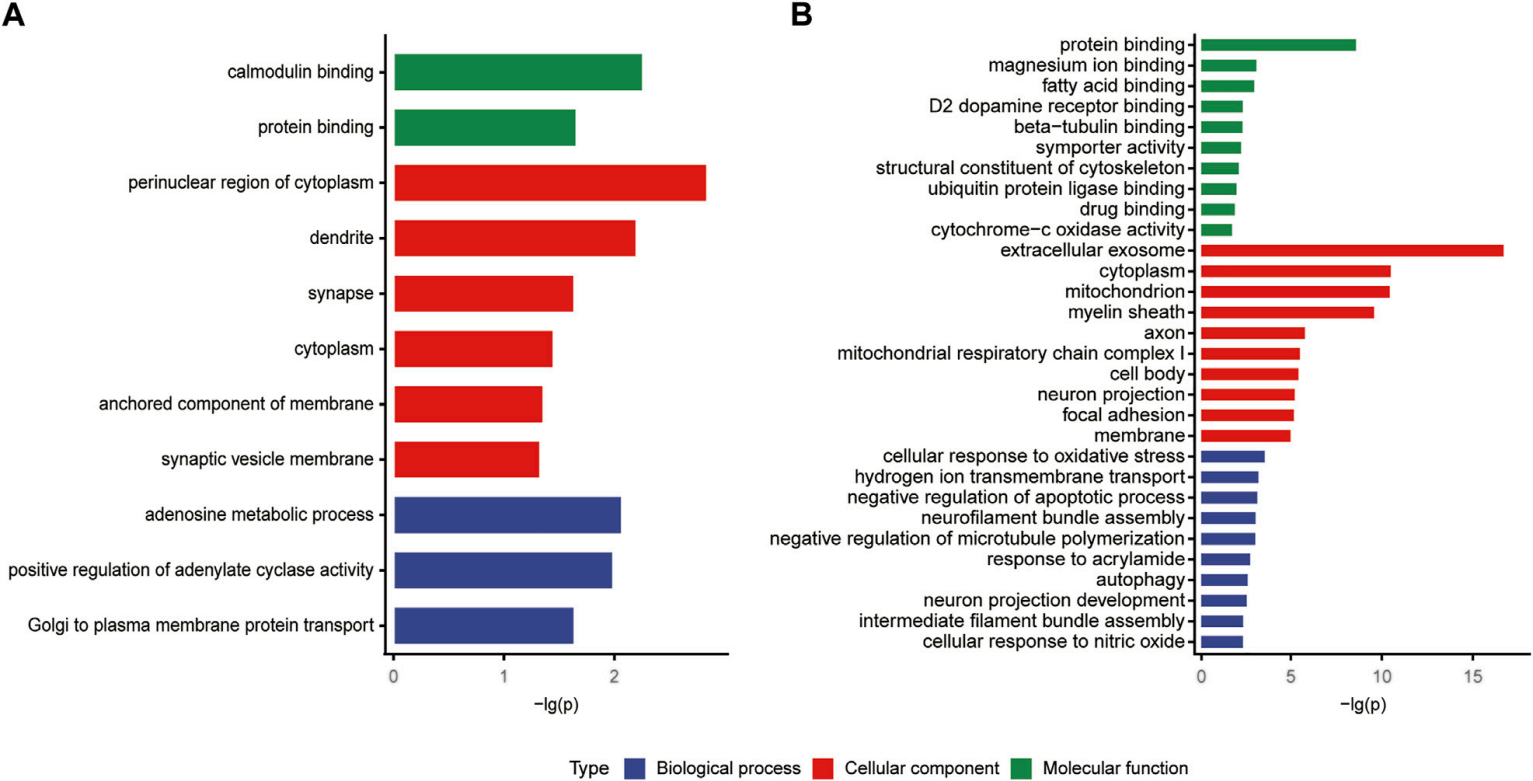
**(A and B)** GO analysis of the 22 Chaihu-Shugan-San advanced proteins and 323 DEPs.

The classifications of the 22 Chaihu-Shugan-San advanced proteins and 323 DEPs were enriched and analyzed to comprehend the importance of certain groups in the GO annotation (Figure 7). In the biological process, 22 Chaihu-Shugan-San advanced proteins acted on adenosine metabolic process (*p* = 0.00852), positive regulation of adenylate cyclase activity (*p* = 0.01022), and Golgi to plasma membrane protein transport (*p* = 0.02286). Synapse was enriched in the cellular component (*p* = 0.02301). Meanwhile, protein binding was enriched in the molecular function (*p* = 0.02189) (Figure 7A). 323 targets acted on the biological process of hydrogen ion transmembrane transport (*p* = 0.00051), response to acrylamide (*p* = 0.00146), and cellular response to nitric oxide (*p* = 0.00364). Extracellular exosome was the most significantly enriched group in the cellular component (*p* = 1.72E-17). Meanwhile, protein binding was the most significant term in the molecular function (*p* = 2.22E-09) (Figure 7B).

#### The Pathways Enrichment Analysis of DEPs

**FIGURE 8:**
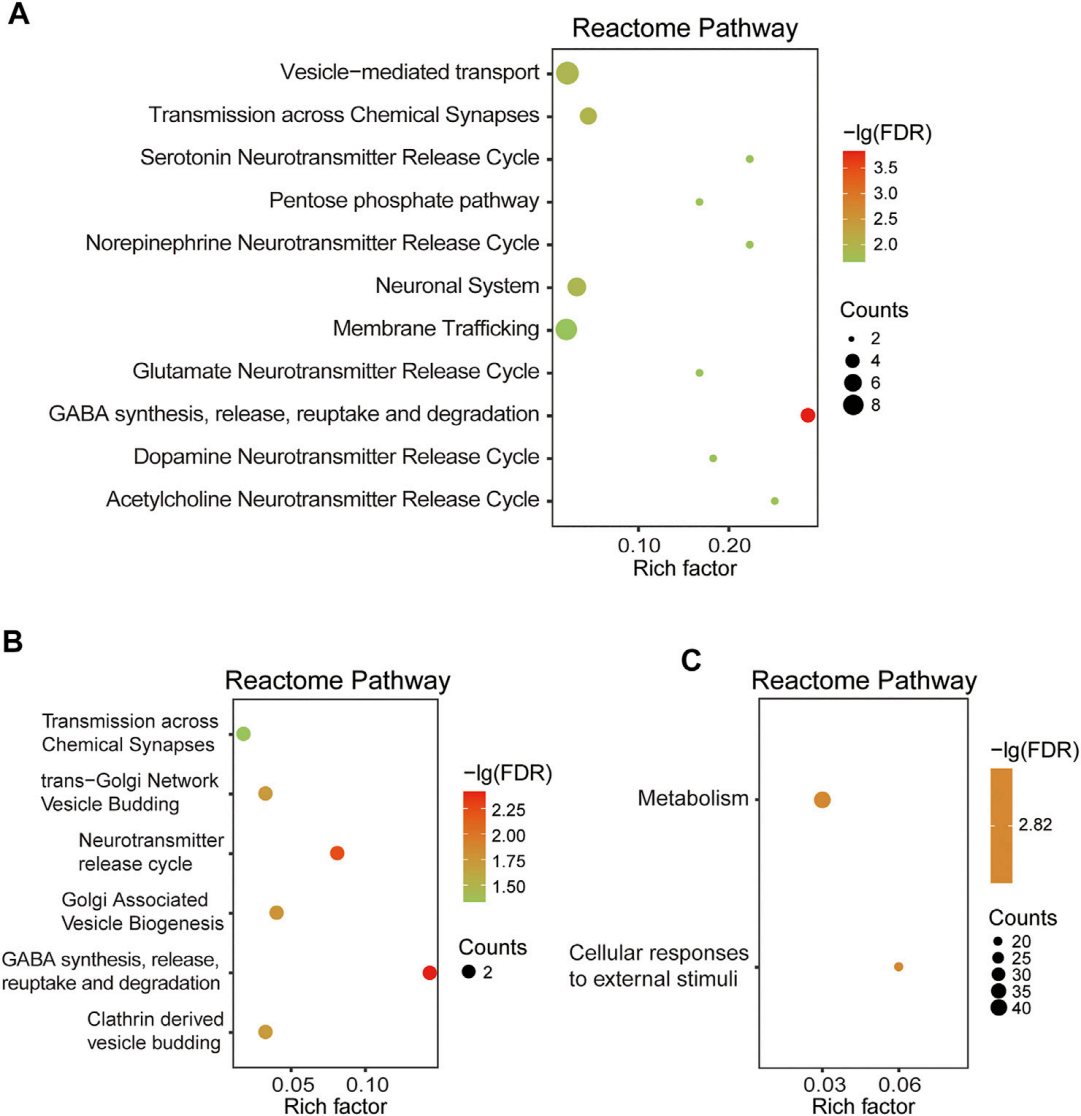
**(A)** RCT analysis of 110 DEPs. **(B)** RCT analysis of 22 DEPs. **(C)** RCT analysis of 323 DEPs.

Based on 110 Chaihu-Shugan-San DEPs, the analysis of pathway enrichment was also carried out. The obtained outcomes showed a total of 11 significantly enriched pathways (Figure 8A and Table 2). The enriched pathways were mainly related to some neurotransmitter’s release and transmission cycle (e.g., γ-aminobutyric acid (GABA), glutamate, serotonin, norepinephrine, dopamine, and acetylcholine). GABA synthesis, release, reuptake, and degradation was the most significant one. Four proteins enriched this pathway that DnaJ homolog subfamily C member 5 (Dnajc5), Glutamate decarboxylase 2 (Gad2), Synaptotagmin-1 (Syt1), and Vesicle-associated membrane protein 2 (Vamp2).

**TABLE 2:** Pathway enrichment analysis of 110 DEPs.

Term ID	Term description	Count	FDR	Genes
RNO-888590	GABA synthesis, release, reuptake and degradation	4	0.00019	Dnajc5,Gad2,Syt1,Vamp2
RNO-112315	Transmission across Chemical Synapses	5	0.0135	Dnajc5,Gad2,Syt1,Tspan7,Vamp2
RNO-112316	Neuronal System	6	0.016	Dnajc5,Gad2,Homer2,Syt1,Tspan7,Vamp2
RNO-5653656	Vesicle-mediated transport	9	0.016	Cnih2,Klc1,Rab9a,Sec13,Sparc,Stx16,Syt1,Tpd52l1,Vamp2
RNO-264642	Acetylcholine Neurotransmitter release Cycle	2	0.0263	Syt1,Vamp2
RNO-181429	Serotonin Neurotransmitter release Cycle	2	0.0275	Syt1,Vamp2
RNO-181430	Norepinephrine Neurotransmitter release Cycle	2	0.0275	Syt1,Vamp2
RNO-199991	Membrane Trafficking	8	0.0275	Cnih2,Klc1,Rab9a,Sec13,Stx16,Syt1,Tpd52l1,Vamp2
RNO-212676	Dopamine Neurotransmitter release Cycle	2	0.0275	Syt1,Vamp2
RNO-210500	Glutamate Neurotransmitter release Cycle	2	0.0287	Syt1,Vamp2
RNO-71336	Pentose phosphate pathway	2	0.0287	Prps2,Rpe

Based on 22 Chaihu-Shugan-San advanced proteins, pathway analysis results identified 6 significantly enriched pathways (Figure 8B and Table 3). The enriched pathways were mainly related to neurotransmitter release cycle (contain GABA) and Golgi-associated vesicle biogenesis. GABA synthesis, release, reuptake, and degradation also was the most significant one. Two proteins enriched this pathway that Gad2 and Vamp2. Based on 323 proteins, only 2 significant pathways were enriched: Metabolism and Cellular responses to external stimuli (Figure 8C and Table 4).

**TABLE 3:** Pathway enrichment analysis of 22 DEPs.

Term ID	Term description	Count	FDR	Genes
RNO-888590	GABA synthesis, release, reuptake and degradation	2	0.0053	Gad2,Vamp2
RNO-112310	Neurotransmitter release cycle	2	0.0077	Gad2,Vamp2
RNO-432722	Golgi Associated Vesicle Biogenesis	2	0.02	Tpd52l1,Vamp2
RNO-199992	trans-Golgi Network Vesicle Budding	2	0.0225	Tpd52l1,Vamp2
RNO-421837	Clathrin derived vesicle budding	2	0.0225	Tpd52l1,Vamp2
RNO-112315	Transmission across Chemical Synapses	2	0.0499	Gad2,Vamp2

**TABLE 4:** Pathway enrichment analysis of 323 DEPs.

Term ID	Term description	Count	FDR	Genes
RNO-1430728	Metabolism	40	0.0015	Abhd14b,Adss,Agpat4,Ak1,Arsa,Asah1,Comt,Dbi,Dnm2,Eno1, Enoph1,Ephx1,Fabp7,Galns,Gcdh,Ggt7,Glrx5,Gm2a,Gmpr2,Gss, Hibch,Itpa,Lias,Mgst3,Ndufa11,Ndufab1,Ndufaf4,Ndufb4,Ndufb6,Ndufb7, Nt5c3a,Nudt12,Pdk3,Pi4k2a,Psma4,Sar1a,Sc5d,Slc23a2, Slco1a5,Tph2
RNO-8953897	Cellular responses to external stimuli	17	0.0015	Actr1a,Capza2,Cdc26,Chmp2b,Cul2,Hif1an,Lamtor1,Map1lc3a, Map3k5,Mt3,Nudt2,Psma4,Rbx1,Rragc,Tceb2,Tubb2a,Tubb2b

#### PPI Analysis of DEPs

**FIGURE 9:**
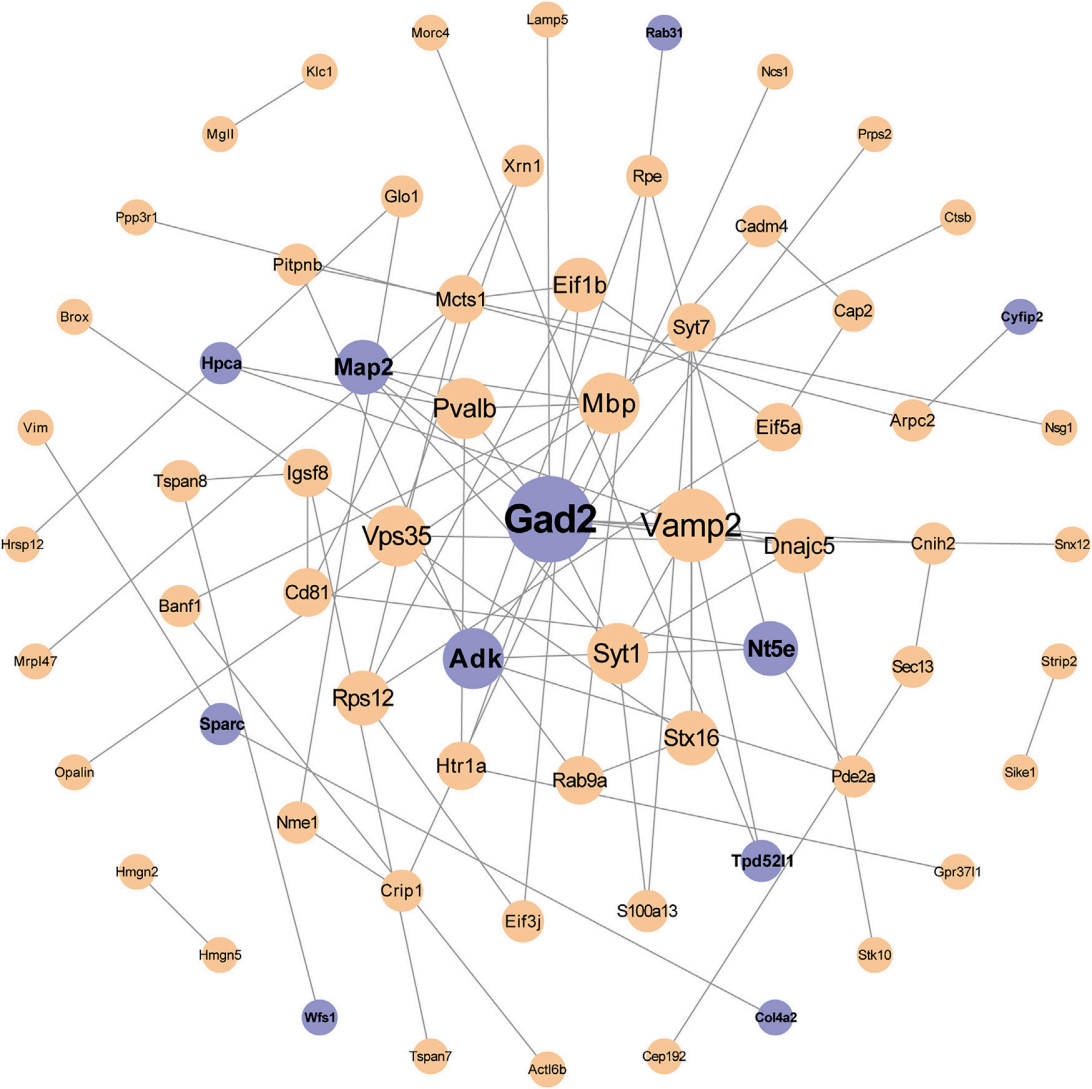
PPI network analyzed by the STRING database and the Cytoscape software. The nodes represent proteins. The size of the node represents the value of degree (a larger size indicates a higher degree). The purple nodes represent the intersection of 110 Chaihu-Shugan-San DEPs (yellow) and 22 Chaihu-Shugan-San advanced proteins.

The PPI network by the STRING database and Cytoscape software was constructed to identify the key targets of Chaihu-Shugan-San against depression. First, 110 DEPs were uploaded to the STRING database and the analysis of the PPI network showed 68 DEPs were interconnected, whilst the other 42 DEPs did not exhibit any category of connection utilizing the default setting. Figure 9 presents a whole perspective of the relationships within 68 targets by Cytoscape software. Additionally, 11 DEPs belong to 22 Chaihu-Shugan-San advanced proteins. According to the degree of proteins, several proteins were key targets in the network, such as Gad2, Vamp2, and Syt1.

#### Validation of DEPs

**FIGURE 10:**
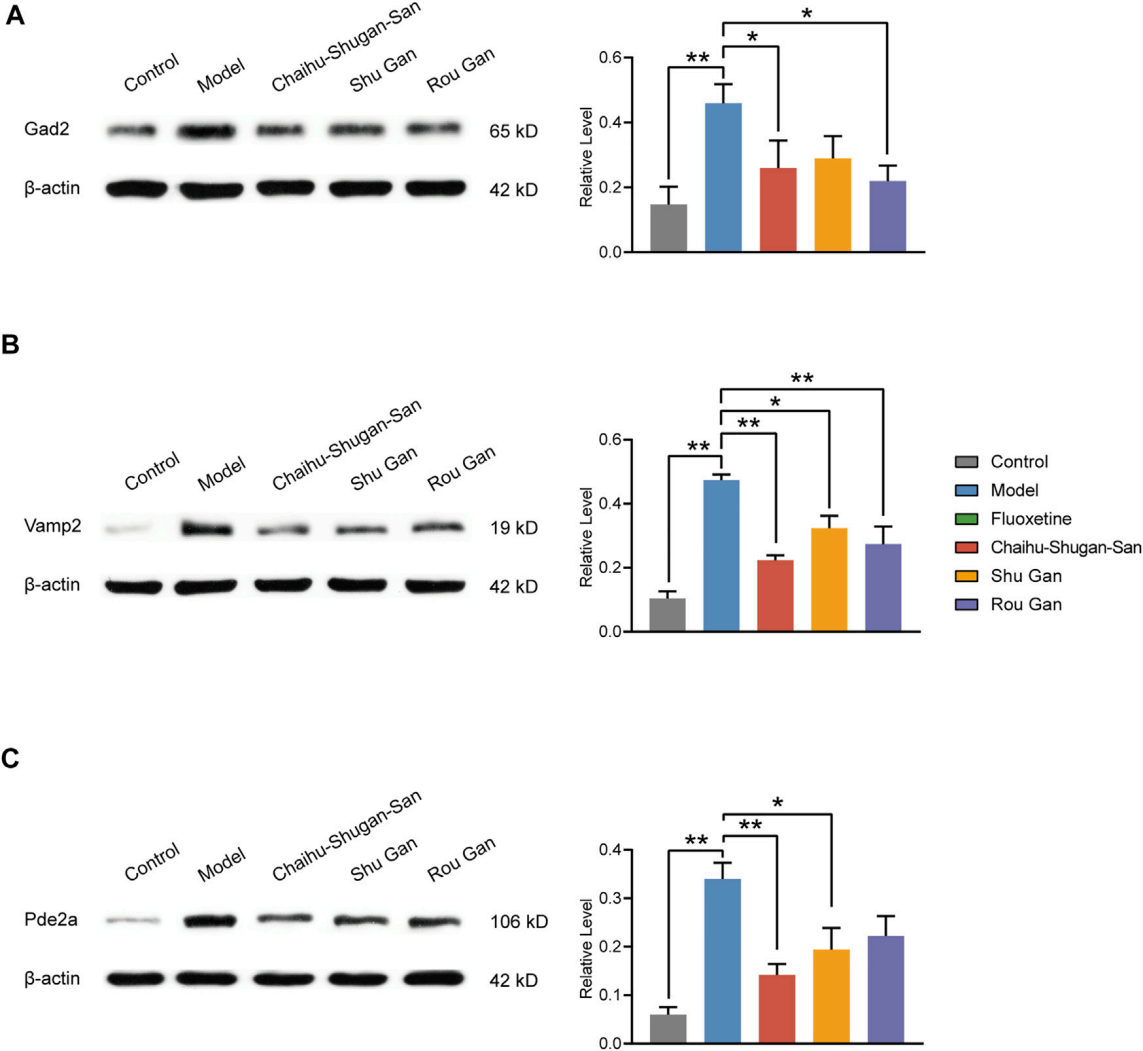
Validation of Gad2 **(A)**, Vamp2 **(B)**, and Pde2a **(C)**. The analysis of western blotting demonstrated the expression levels of the Control, Model, Chaihu-Shugan-San, Shu Gan, and Rou Gan group in the rats’ hippocampus. (*n* = 5, ∗*p* < 0.05, ∗∗*p* < 0.01, data are presented as mean ± SEM).

Western blotting was executed to further validate the candidate DEPs containing Gad2, Vamp2, and Pde2a. As displayed in Figure 10A (F_4,20_ = 5.253, *p* < 0.01), Chaihu-Shugan-San and Rou Gan treatment down-regulated Gad2 significantly in comparison with the model group (*p* < 0.05). As displayed in Figure 10B (F_4,20_ = 16.908, *p* < 0.01), Chaihu-Shugan-San, Shu Gan, and Rou Gan treatment down-regulated Vamp2 significantly in comparison with the model group (*p* < 0.05). As displayed in Figure 10C (F_4,20_ = 9.652, *p* < 0.01), Chaihu-Shugan-San and Shu Gan treatment down-regulated Pde2a significantly in comparison with the model group (*p* < 0.05).

## Discussion

In TCM theory, the Gan is the center of regulating emotions (Scheid, 2013; Li XJ. et al., 2020). Chaihu-Shugan-San consists of Shu Gan (stagnated Gan-Qi dispersion) and Rou Gan (Gan nourishment to alleviate pain), treating complex emotional diseases such as depression (Qiu et al., 2014b). Modern studies have also shown that Chaihu-Shugan-San can inhibit apoptosis in liver cells and participate in phospholipid and bile acid metabolism to counteract CUMS-induced liver injury in rats (Jia et al., 2017). While for the hippocampus, which is responsible for emotion regulation (Snyder et al., 2011), a comprehensive exploration is lacking. The anti-depressive mechanisms and the compatibility advantage of Chaihu-Shugan-San in the hippocampus of CUMS-induced rats were explored by proteomics for the first time in this study.

After a 2-weeks treatment of Chaihu-Shugan-San or its decomposed recipes, behavioral data including SPT and FST demonstrated that Chaihu-Shugan-San exhibited better therapeutic effects in the CUMS models. Our proteomics data showed that Chaihu-Shugan-San controlled 110 DEPs. By mapping the PPI network of 110 DEPs, we found that Gad2 and Vamp2 were the key targets in the network. Bioinformatics analysis suggested that the signalling pathways affected by Chaihu-Shugan-San contained neurotransmitters release and transmit pathways (e.g., GABA, glutamate, serotonin, norepinephrine, dopamine, and acetylcholine), which play an important role in the pathogenesis of depression (Kraus et al., 2017; Murrough et al., 2017; Gunduz-Bruce et al., 2019). Furthermore, the 22 Chaihu-Shugan-San advanced proteins which were mainly enriched in GABA release cycle was additionally changed by Chaihu-Shugan-San treatment. The 323 proteins whose expression was changed by Shu Gan or Rou Gan treatment alone were enriched in metabolism and cellular responses to external stimuli. Finally, we used molecular biotechnology to verify the credibility of quantitative proteomics by selected proteins from DEPs including Gad2, Vamp2, and Pde2a.

TCM has extraordinary therapeutic effects owing to its complicated mechanisms, in which the compatibility of its recipes counts a great deal. Chaihu-Shugan-San, a combination of Shu Gan and Rou Gan, has been widely used as a classical antidepressant formula in TCM practice (Qiu et al., 2014b). Weight changes and appetite changes are common symptoms of depression (Fava, 2000). After 4 weeks of CUMS procedures, the bodyweight growth and food intake of the stressed rats were significantly less than the unstressed rats. Chaihu-Shugan-San or Shu Gan could increase the bodyweight growth and food intake of CUMS-induced rats, exerting a fluoxetine-like effect. We used SPT and FST to assess the antidepressant efficacy of Chaihu-Shugan-San, Shu Gan, and Rou Gan in CUSM rats. SPT evaluates sensitivity to reward. A decreased preference for consumption of sucrose reflects anhedonia (lose pleasure in rewards) that is the core symptom of depression (Moreau, 2002). In SPT, Chaihu-Shugan-San significantly improves the sucrose preference index, which is similar to fluoxetine. Neither Shu Gan nor Rou Gan increases the sucrose preference index significantly. FST is the gold standard for despair testing (Becker et al., 2021). FST assesses learned helplessness, which is a feature of depression-like behavior (Yankelevitch-Yahav et al., 2015). In FST, the Chaihu-Shugan-San and Shu Gan groups significantly lower the immobility time, which is similar to fluoxetine. CUMS-induced rats showed lacked pleasure, desperate behavior, loss of appetite, and impaired weight growth, similar to human depressive disorder (Krishnan et al., 2007; Yang et al., 2017; Xing et al., 2019). The experimental results indicated that the depression model was successfully established. After 2 weeks of treatment, all behavioral disturbances created via CUMS were correspondingly reversed, indicating that Chaihu-Shugan-San and fluoxetine possessed anti-depressive impacts. Chaihu-Shugan-San exhibited better therapeutic effects in the CUMS models as compared with its decomposed recipes.

Then, to further analyze the advantage of TCM compatibility and explore the anti-depression mechanisms of Chaihu-Shugan-San, we used quantitative proteomics and bioinformatics analysis to identify and analyze DEPs. Chaihu-Shugan-San, Shu Gan, and Rou Gan regulated 110, 12, and 407 DEPs, respectively. Although the targets of Chaihu-Shugan-San are less than the sum of Shu Gan and Rou Gan, bioinformatics analysis shows that it may affect the classic antidepressant pathway (e.g., GABA, glutamate, serotonin, norepinephrine, dopamine, and acetylcholine pathway) (Kraus et al., 2017; Murrough et al., 2017; Gunduz-Bruce et al., 2019). The GABA synthesis, release, reuptake, and degradation was the most significant pathway by 110 DEPs enriched. Compared with Shu Gan or Rou Gan alone, the expression of 22 advanced proteins was additionally changed by Chaihu-Shugan-San treatment, whereas the expression of 323 proteins whose expression was changed by Shu Gan or Rou Gan alone were not changed by Chaihu-Shugan-San treatment. Interestingly, 22 DEPs focus on GABA synthesis, release, reuptake, and degradation pathway-one of the key pathways of Chaihu-Shugan-San to treat depression. The signalling pathways regulated by Shu Gan are the transport of small molecules (cations, anions, amino acids, and oligopeptides) (Supplementary Figure S4A). The signalling pathways regulated by Rou Gan are metabolism, cellular responses to external stimuli, and transport to the Golgi (Supplementary Figure S4B). Meanwhile, based on 323 proteins, only 2 pathways were enriched: metabolism and cellular responses to external stimuli. These 323 proteins that are not enriched in the key pathway of depression are targeted by Shu Gan or Rou Gan. Accordingly, the compatibility of prescriptions is not simply increasing the number of targets, but increasing the accuracy of targets. This explains why Shu Gan and Rou Gan did not achieve the antidepressant effect of Chaihu-Shugan-San.

Our data show that Chaihu-Shugan-San treats depression potentially via influence 110 DEPs (Gad2, Vamp2, Pde2a, etc.), and neurotransmitters release and transmission cycle (e.g., GABA, glutamate, serotonin, norepinephrine, dopamine, and acetylcholine). Compared with Shu Gan and Rou Gan, the 22 Chaihu-Shugan-San advanced proteins and the affected GABA pathway may be the advantages of Chaihu-Shugan-San compatibility. Remarkably, the PPI of Chaihu-Shugan-San’s targets showed a closely linked network, Gad2 and Vamp2 are key targets in the network. Gad2 and Vamp2 also are enriched in the GABA and glutamate release pathways. GABA and glutamate are the primary inhibitory and excitatory neurotransmitters in the brain. An impaired balance of neural excitation and inhibition is one of the pathological characteristics of depression (Abdallah et al., 2014; Fee et al., 2017). Stress leads to activation of the HPA axis. Then it results in the over-release of glucocorticoid, dysfunction of glucocorticoid receptors, and subsequent the disorder of glutamate transmission and an excessive concentration of glutamate in the synaptic space. This could create excitatory toxicity to neurons, resulting in neuron degeneration, aging, and death, finally resulting in mental disorders, for instance, depression (Ferrer et al., 2018). Glutamate levels are elevated in the brains with depression in patients or rats (Hashimoto et al., 2007; De Vasconcellos-Bittencourt et al., 2011). Glutamate release inhibitors have antidepressant properties and are used in clinics widely (Deutschenbaur et al., 2016; Lener et al., 2017). Activation of the HPA axis also changes the expression of postsynaptic GABA_A_ receptors, then reduces GABA release in presynaptic (Luscher et al., 2011; Maguire, 2018). The studies from depression subjects indicate the GABAergic deficit in the central nervous system (Croarkin et al., 2011; Möhler, 2012). The enhancers of GABA_A_ receptors have been used as antidepressants (Papakostas and Shelton, 2008; Morishita, 2009). Our previous research demonstrates that the components of Chaihu-Shugan-San play an antidepressant role by regulating the HPA axis and inhibiting neurotransmitter reuptakes (e.g., norepinephrine, serotonin, and dopamine) (Zhang et al., 2011). Therefore, we speculate that Chaihu-Shugan-San affects glutamate and GABA signalling pathways to treat depression via the HPA axis. Of course, the concrete anti-depressive mechanism needs to further explore.

We chose Gad2, Vamp2, and Pde2a for western blotting. Gad2 belongs to the group II glutamate decarboxylase family that catalyzes the decarboxylation of glutamate to produce GABA. Glutamate regulates the expression of Gad2 by the autocrine effect on sensory terminal BDNF, and depletion of glutamate release lower levels of BDNF and Gad2 (Mende et al., 2016). Gad2 activity underlies enhanced release of GABA at high frequencies of stimulation, showed that this enzyme expression could regulate the efficiency of inhibitory synaptic signalling (Betley et al., 2009). However, no simple parallel exists between Gad2 and GABA content (Sonnewald et al., 2004; Benagiano et al., 2005; Freichel et al., 2006). Depression patients had Gad2 antibody positive and Gad2 mRNA expression increased (Bowers et al., 1998; Kruse et al., 2015). Our proteomics data and western blotting data show that Gad2 levels in CUMS rats were significantly increased, while the treatment of Chaihu-Shugan-San or its decomposed recipes reversed it. Interestingly, Gad2 is an indicator of diabetes (Atkinson and Eisenbarth, 2001). Ample clinical evidence shows that diabetes is associated with depression, but the internal relationship is unclear (Anderson et al., 2001; Knol et al., 2006; Mezuk et al., 2008). This finding may provide clues for depression complicated with diabetes.

Vamp2, a core soluble N-ethylmaleimide-sensitive factor attachment protein receptor (SNARE) protein residing on synaptic vesicles, forms helix bundles with SNAP25 and syntaxin-1 for the SNARE assembly (Ramakrishnan et al., 2012; Wang et al., 2020). SNARE proteins were the main neurotransmitter release-associated proteins involving in the release procedure and synaptic vesicle fusion (Kiessling et al., 2013; Stratton et al., 2016). It is the crucial factor that mediates the complicated interactions between the presynaptic membrane and vesicle fusion. According to our proteomics and western blotting data, the Vamp2 expression level was up-regulated in CUMS rats, while treatment of Chaihu-Shugan-San and its decomposed recipes could counteract the change significantly. SNARE protein complex formation is considerably enhanced during depression (Katrancha and Koleske, 2015; Cao et al., 2018). Furthermore, antidepressant reboxetine, fluoxetine, and desipramine could remarkably diminish SNARE complex expression (Bonanno et al., 2005). A previous study has illustrated that acupuncture could reduce the expression of SNARE protein (Vamp2) in depressive animals (Fan et al., 2016). A recent study shows that anti-depressive TCM-paeoniflorin noticeably reduced hippocampal glutamate via inhibiting the level of SNARE complex and Vamp2 (Li YC. et al., 2020).

Pde2a, an enzyme that catalyzes the hydrolysis of cyclic guanosine monophosphate (cGMP) and cyclic adenosine monophosphate (cAMP), may become a potential pharmacological target for neuropsychiatric diseases (Conti and Beavo, 2007; Knott et al., 2017). Proteomics data and western blotting results showed Pde2a was decreased after Chaihu-Shugan-San or Chaihu-Shugan-San’s decomposed recipes treatment. Pde2a is a crucial point of compartmentalized cross-talk between cGMP and cAMP signalling (Reierson et al., 2011). cGMP/cAMP signalling pathway, being generally anti-inflammatory, could help to reduce oxidative stress (Masood et al., 2008). Inhibition of cGMP is widespread across various antidepressants such as ketamine (Li et al., 2006; Cunha et al., 2015). A study showed Pde2a mRNA expression increased in the depression cell model (Zhu et al., 2019). The most commonly characterized selective Pde2a inhibitor, namely Bay 60–7550, generates antidepressant efficacy in the depression animal model (Masood et al., 2009; Xu et al., 2013; Ding et al., 2014). Pde2a inhibitor had significant neuroprotective effects on depression by affecting the cGMP and cAMP signalling pathways, contain increasing the ratio of the expression of pCREB/CREB and BDNF (Liu L. et al., 2018).

Taken together, the curative effect of Chaihu-Shugan-San is better than those of Shu Gan and Rou Gan in behavioral evaluation, and Chaihu-Shugan-San affects the classic antidepressant pathway more focused than Shu Gan and Rou Gan in proteomics. This study suggests that Gad2, Vamp2, and Pde2a are potentially involved in depression remission treated by Chaihu-Shugan-San. Therefore, these proteins might be the antidepression targets of Chaihu-Shugan-San. Nevertheless, the present study had several limitations. First, we are still at the beginning of our understanding on how these proteins are operated and how they are regulated by Chaihu-Shugan-San. Second, modeling the full complexity of the human disorder in animals is difficult, especially mood disorders such as depression. Tests on assessing animal models of depression also lack the mechanistic specificity to be used universally to elucidate the neurobiological mechanisms of depression in humans. Data from human samples need to be supplemented in future studies.

## Conclusion

In this study, we provide proteomic clues to explore the merit of prescription compatibility in Chaihu-Shugan-San and explores mechanisms of Chaihu-Shugan-San anti-depression. Our research reveals that Chaihu-Shugan-San treats depression via multiple targets and pathways, which may include regulations of 110 DEPs and some neurotransmitter’s transmission cycle (e.g., GABA, glutamate, serotonin, norepinephrine, dopamine, and acetylcholine). Compared with Shu Gan and Rou Gan, the 22 Chaihu-Shugan-San advanced proteins and the affected GABA pathway may be the advantages of Chaihu-Shugan-San compatibility. Those findings would contribute to revealing the prescription combination advantages of Chaihu-Shugan-San for antidepression, and also a rational way for clarifying the composition rules of TCM. Meanwhile, this research offers data and theory support for the clinical application of Chaihu-Shugan-San.

## Data Availability

The original contributions presented in the study are publicly available. This data can be found here: ProteomeXchange via the PRIDE database, with the accession number PXD024436.

## References

[B1] Abdallah C. G. Jiang L. De Feyter H. M. Fasula M. Krystal J. H. Rothman D. L. (2014). Glutamate Metabolism in Major Depressive Disorder. Am. J. Psychiatry 171, 1320–1327. 10.1176/appi.ajp.2014.14010067 25073688 PMC4472484

[B2] Anderson R. J. Freedland K. E. Clouse R. E. Lustman P. J. (2001). The Prevalence of Comorbid Depression in Adults with Diabetes: a Meta-Analysis. Diabetes Care 24, 1069–1078. 10.2337/diacare.24.6.1069 11375373

[B3] Antoniuk S. Bijata M. Ponimaskin E. Wlodarczyk J. (2019). Chronic Unpredictable Mild Stress for Modeling Depression in Rodents: Meta-Analysis of Model Reliability. Neurosci. Biobehav Rev. 99, 101–116. 10.1016/j.neubiorev.2018.12.002 30529362

[B4] Atkinson M. A. Eisenbarth G. S. (2001). Type 1 Diabetes: New Perspectives on Disease Pathogenesis and Treatment. Lancet 358, 221–229. 10.1016/s0140-6736(01)05415-0 11476858

[B5] Becker M. Pinhasov A. Ornoy A. (2021). Animal Models of Depression: What Can They Teach Us about the Human Disease? Diagnostics 11, 123. 10.3390/diagnostics11010123 33466814 PMC7830961

[B6] Benagiano V. Lorusso L. Coluccia A. Tarullo A. Flace P. Girolamo F. (2005). Glutamic Acid Decarboxylase and GABA Immunoreactivities in the Cerebellar Cortex of Adult Rat after Prenatal Exposure to a Low Concentration of Carbon Monoxide. Neuroscience 135, 897–905. 10.1016/j.neuroscience.2005.06.058 16112480

[B7] Betley J. N. Wright C. V. Kawaguchi Y. Erdélyi F. Szabó G. Jessell T. M. (2009). Stringent Specificity in the Construction of a GABAergic Presynaptic Inhibitory Circuit. Cell 139, 161–174. 10.1016/j.cell.2009.08.027 19804761 PMC2812434

[B8] Bonanno G. Giambelli R. Raiteri L. Tiraboschi E. Zappettini S. Musazzi L. (2005). Chronic Antidepressants Reduce Depolarization-Evoked Glutamate Release and Protein Interactions Favoring Formation of SNARE Complex in hippocampus. J. Neurosci. 25, 3270–3279. 10.1523/jneurosci.5033-04.2005 15800181 PMC6724889

[B9] Bowers G. Cullinan W. E. Herman J. P. (1998). Region-specific Regulation of Glutamic Acid Decarboxylase (GAD) mRNA Expression in central Stress Circuits. J. Neurosci. 18, 5938–5947. 10.1523/jneurosci.18-15-05938.1998 9671680 PMC6793042

[B10] Cao Y. J. Wang Q. Zheng X. X. Cheng Y. Zhang Y. (2018). Involvement of SNARE Complex in the hippocampus and Prefrontal Cortex of Offspring with Depression Induced by Prenatal Stress. J. Affect Disord. 235, 374–383. 10.1016/j.jad.2018.04.053 29674253

[B11] Chen X. Q. Li C. F. Chen S. J. Liang W. N. Wang M. Wang S. S. (2018). The Antidepressant-like Effects of Chaihu Shugan San: Dependent on the Hippocampal BDNF-TrkB-ERK/Akt Signaling Activation in Perimenopausal Depression-like Rats. Biomed. Pharmacother. 105, 45–52. 10.1016/j.biopha.2018.04.035 29843044

[B12] Chi X. Wang S. Baloch Z. Zhang H. Li X. Zhang Z. (2019). Research Progress on Classical Traditional Chinese Medicine Formula Lily Bulb and Rehmannia Decoction in the Treatment of Depression. Biomed. Pharmacother. 112, 108616. 10.1016/j.biopha.2019.108616 30780102

[B13] Conti M. Beavo J. (2007). Biochemistry and Physiology of Cyclic Nucleotide Phosphodiesterases: Essential Components in Cyclic Nucleotide Signaling. Annu. Rev. Biochem. 76, 481–511. 10.1146/annurev.biochem.76.060305.150444 17376027

[B14] Croarkin P. E. Levinson A. J. Daskalakis Z. J. (2011). Evidence for GABAergic Inhibitory Deficits in Major Depressive Disorder. Neurosci. Biobehav Rev. 35, 818–825. 10.1016/j.neubiorev.2010.10.002 20946914

[B15] Cunha M. P. Pazini F. L. Ludka F. K. Rosa J. M. Oliveira Á. Budni J. (2015). The Modulation of NMDA Receptors and L-Arginine/nitric Oxide Pathway Is Implicated in the Anti-immobility Effect of Creatine in the Tail Suspension Test. Amino Acids 47, 795–811. 10.1007/s00726-014-1910-0 25555469

[B16] De Vasconcellos-Bittencourt A. P. Vendite D. A. Nassif M. Crema L. M. Frozza R. Thomazi A. P. (2011). Chronic Stress and Lithium Treatments Alter Hippocampal Glutamate Uptake and Release in the Rat and Potentiate Necrotic Cellular Death after Oxygen and Glucose Deprivation. Neurochem. Res. 36, 793–800. 10.1007/s11064-011-0404-7 21253855

[B17] Deutschenbaur L. Beck J. Kiyhankhadiv A. Mühlhauser M. Borgwardt S. Walter M. (2016). Role of Calcium, Glutamate and NMDA in Major Depression and Therapeutic Application. Prog. Neuropsychopharmacol. Biol. Psychiatry 64, 325–333. 10.1016/j.pnpbp.2015.02.015 25747801

[B18] Ding L. Zhang C. Masood A. Li J. Sun J. Nadeem A. (2014). Protective Effects of Phosphodiesterase 2 Inhibitor on Depression- and Anxiety-like Behaviors: Involvement of Antioxidant and Anti-apoptotic Mechanisms. Behav. Brain Res. 268, 150–158. 10.1016/j.bbr.2014.03.042 24694839 PMC4075066

[B19] Duman R. S. Aghajanian G. K. (2012). Synaptic Dysfunction in Depression: Potential Therapeutic Targets. Science 338, 68–72. 10.1126/science.1222939 23042884 PMC4424898

[B20] Fan L. Chen Z. Fu W. Xu N. Liu J. Lu A. (2016). Soluble N-Ethylmaleimide-Sensitive Factor Attachment Receptor (SNARE) Protein Involved in the Remission of Depression by Acupuncture in Rats. J. Acupunct Meridian Stud. 9, 242–249. 10.1016/j.jams.2016.04.002 27776762

[B21] Fava M. (2000). Weight Gain and Antidepressants. J. Clin. Psychiatry 61 (Suppl. 11), 37–41. 10.4088/jcp.v61n1109 10926053

[B22] Fee C. Banasr M. Sibille E. (2017). Somatostatin-Positive Gamma-Aminobutyric Acid Interneuron Deficits in Depression: Cortical Microcircuit and Therapeutic Perspectives. Biol. Psychiatry 82, 549–559. 10.1016/j.biopsych.2017.05.024 28697889 PMC5610074

[B23] Ferrer A. Costas J. Labad J. Salvat-Pujol N. Segalàs C. Urretavizcaya M. (2018). FKBP5 Polymorphisms and Hypothalamic-Pituitary-Adrenal axis Negative Feedback in Major Depression and Obsessive-Compulsive Disorder. J. Psychiatr. Res. 104, 227–234. 10.1016/j.jpsychires.2018.08.003 30107269

[B24] Freichel C. Potschka H. Ebert U. Brandt C. Löscher W. (2006). Acute Changes in the Neuronal Expression of GABA and Glutamate Decarboxylase Isoforms in the Rat Piriform Cortex Following Status Epilepticus. Neuroscience 141, 2177–2194. 10.1016/j.neuroscience.2006.05.040 16797850

[B25] Gunduz-Bruce H. Silber C. Kaul I. Rothschild A. J. Riesenberg R. Sankoh A. J. (2019). Trial of SAGE-217 in Patients with Major Depressive Disorder. N. Engl. J. Med. 381, 903–911. 10.1056/NEJMoa1815981 31483961

[B26] Hashimoto K. Sawa A. Iyo M. (2007). Increased Levels of Glutamate in Brains from Patients with Mood Disorders. Biol. Psychiatry 62, 1310–1316. 10.1016/j.biopsych.2007.03.017 17574216

[B27] Haw R. Stein L. (2012). Using the Reactome Database. Curr. Protoc. Bioinform. 38, 1–23. 10.1002/0471250953.bi0807s38 PMC3427849 22700314

[B28] Hu C. Luo Y. Wang H. Kuang S. Liang G. Yang Y. (2017). Re-evaluation of the Interrelationships Among the Behavioral Tests in Rats Exposed to Chronic Unpredictable Mild Stress. PLoS One 12, e0185129. 10.1371/journal.pone.0185129 28931086 PMC5607203

[B29] Jassal B. Matthews L. Viteri G. Gong C. Lorente P. Fabregat A. (2020). The Reactome Pathway Knowledgebase. Nucleic Acids Res. 48, D498–d503. 10.1093/nar/gkz1031 31691815 PMC7145712

[B30] Jia H. M. Yu M. Ma L. Y. Zhang H. W. Zou Z. M. (2017). Chaihu-Shu-Gan-San Regulates Phospholipids and Bile Acid Metabolism against Hepatic Injury Induced by Chronic Unpredictable Stress in Rat. J. Chromatogr. B Analyt Technol. Biomed. Life Sci. 1064, 14–21. 10.1016/j.jchromb.2017.08.003 28886478

[B31] Katrancha S. M. Koleske A. J. (2015). SNARE Complex Dysfunction: A Unifying Hypothesis for Schizophrenia. Biol. Psychiatry 78, 356–358. 10.1016/j.biopsych.2015.07.013 26296424 PMC4703341

[B32] Kiessling V. Ahmed S. Domanska M. K. Holt M. G. Jahn R. Tamm L. K. (2013). Rapid Fusion of Synaptic Vesicles with Reconstituted Target SNARE Membranes. Biophys. J. 104, 1950–1958. 10.1016/j.bpj.2013.03.038 23663838 PMC3647153

[B33] Kim S. H. Han J. Seog D. H. Chung J. Y. Kim N. Hong Park Y. (2005). Antidepressant Effect of Chaihu-Shugan-San Extract and its Constituents in Rat Models of Depression. Life Sci. 76, 1297–1306. 10.1016/j.lfs.2004.10.022 15642599

[B34] Knol M. J. Twisk J. W. Beekman A. T. Heine R. J. Snoek F. J. Pouwer F. (2006). Depression as a Risk Factor for the Onset of Type 2 Diabetes Mellitus. A Meta-Analysis. Diabetologia 49, 837–845. 10.1007/s00125-006-0159-x 16520921

[B35] Knott E. P. Assi M. Rao S. N. Ghosh M. Pearse D. D. (2017). Phosphodiesterase Inhibitors as a Therapeutic Approach to Neuroprotection and Repair. Int. J. Mol. Sci. 18, 696. 10.3390/ijms18040696 PMC5412282 28338622

[B36] Kraus C. Castrén E. Kasper S. Lanzenberger R. (2017). Serotonin and Neuroplasticity - Links between Molecular, Functional and Structural Pathophysiology in Depression. Neurosci. Biobehav Rev. 77, 317–326. 10.1016/j.neubiorev.2017.03.007 28342763

[B37] Krishnan V. Han M. H. Graham D. L. Berton O. Renthal W. Russo S. J. (2007). Molecular Adaptations Underlying Susceptibility and Resistance to Social Defeat in Brain Reward Regions. Cell 131, 391–404. 10.1016/j.cell.2007.09.018 17956738

[B38] Kruse J. L. Lapid M. I. Lennon V. A. Klein C. J. Toole O. O. Pittock S. J. (2015). Psychiatric Autoimmunity: N-Methyl-D-Aspartate Receptor IgG and beyond. Psychosomatics 56, 227–241. 10.1016/j.psym.2015.01.003 25975857

[B39] Lener M. S. Kadriu B. Zarate C. A. Jr. (2017). Ketamine and beyond: Investigations into the Potential of Glutamatergic Agents to Treat Depression. Drugs 77, 381–401. 10.1007/s40265-017-0702-8 28194724 PMC5342919

[B40] Li S.-Q. Su Z.-H. Peng J.-B. Zou Z.-M. Yu C.-Y. (2010). *In Vitro* and *In Vivo* Antioxidant Effects and the Possible Relationship between the Antidepression Efficacy of Traditional Chinese Medicine Formulation Chaihu Shugan San. Chin. J. Nat. Medicines 8, 353–361. 10.1016/s1875-5364(10)60042-8

[B41] Li X. J. Qiu W. Q. Da X. L. Hou Y. J. Ma Q. Y. Wang T. Y. (2020). A Combination of Depression and Liver Qi Stagnation and Spleen Deficiency Syndrome Using a Rat Model. Anat. Rec. (Hoboken) 303, 2154–2167. 10.1002/ar.24388 32353209

[B42] Li X. M. Tanaka K. Sun J. Filipski E. Kayitalire L. Focan C. (2005). Preclinical Relevance of Dosing Time for the Therapeutic index of Gemcitabine-Cisplatin. Br. J. Cancer 92, 1684–1689. 10.1038/sj.bjc.6602564 15841076 PMC2362038

[B43] Li Y. C. Zheng X. X. Xia S. Z. Li Y. Deng H. H. Wang X. (2020). Paeoniflorin Ameliorates Depressive-like Behavior in Prenatally Stressed Offspring by Restoring the HPA axis- and Glucocorticoid Receptor- Associated Dysfunction. J. Affect Disord. 274, 471–481. 10.1016/j.jad.2020.05.078 32663978

[B44] Li Y. F. Zhang Y. Z. Liu Y. Q. Wang H. L. Cao J. B. Guan T. T. (2006). Inhibition of N-Methyl-D-Aspartate Receptor Function Appears to Be One of the Common Actions for Antidepressants. J. Psychopharmacol. 20, 629–635. 10.1177/0269881106059692 16401669

[B45] Li Y. H. Zhang C. H. Qiu J. Wang S. E. Hu S. Y. Huang X. (2014). Antidepressant-like Effects of Chaihu-Shugan-San via SAPK/JNK Signal Transduction in Rat Models of Depression. Pharmacogn Mag. 10, 271–277. 10.4103/0973-1296.137367 25210314 PMC4159920

[B46] Li Y. Zhang C. H. Wang S. E. Qiu J. Hu S. Y. Xiao G. L. (2009). Effects of Chaihu Shugan San on Behavior and Plasma Levels of Corticotropin Releasing Hormone and Adrenocorticotropic Hormone of Rats with Chronic Mild Unpredicted Stress Depression. Zhong Xi Yi Jie He Xue Bao 7, 1073–1077. J. Chin. Integr. Med. 10.3736/jcim20091110 19912741

[B47] Liu L. Zheng J. Huang X. F. Zhu X. Ding S. M. Ke H. M. (2018). The Neuroprotective and Antidepressant-like Effects of Hcyb1, a Novel Selective PDE2 Inhibitor. CNS Neurosci. Ther. 24, 652–660. 10.1111/cns.12863 29704309 PMC6489804

[B48] Liu Q. Sun N. N. Wu Z. Z. Fan D. H. Cao M. Q. (2018). Chaihu-Shugan-San Exerts an Antidepressive Effect by Downregulating miR-124 and Releasing Inhibition of the MAPK14 and Gria3 Signaling Pathways. Neural Regen. Res. 13, 837–845. 10.4103/1673-5374.232478 29863014 PMC5998613

[B49] Liu Y. Wang W. Chen Y. Yan H. Wu D. Xu J. (2020). Simultaneous Quantification of Nine Components in the Plasma of Depressed Rats after Oral Administration of Chaihu-Shugan-San by Ultra-performance Liquid Chromatography/quadrupole-Time-Of-Flight Mass Spectrometry and its Application to Pharmacokinetic Studies. J. Pharm. Biomed. Anal. 186, 113310. 10.1016/j.jpba.2020.113310 32348951

[B50] Luscher B. Shen Q. Sahir N. (2011). The GABAergic Deficit Hypothesis of Major Depressive Disorder. Mol. Psychiatry 16, 383–406. 10.1038/mp.2010.120 21079608 PMC3412149

[B51] Maguire J. (2018). The Relationship between GABA and Stress: 'it's Complicated'. J. Physiol. 596, 1781–1782. 10.1113/jp275937 29524241 PMC5978385

[B52] Malhi G. S. Mann J. J. (2018). Depression. Lancet 392, 2299–2312. 10.1016/s0140-6736(18)31948-2 30396512

[B53] Masood A. Huang Y. Hajjhussein H. Xiao L. Li H. Wang W. (2009). Anxiolytic Effects of Phosphodiesterase-2 Inhibitors Associated with Increased cGMP Signaling. J. Pharmacol. Exp. Ther. 331, 690–699. 10.1124/jpet.109.156729 19684253 PMC2775258

[B54] Masood A. Nadeem A. Mustafa S. J. O'donnell J. M. (2008). Reversal of Oxidative Stress-Induced Anxiety by Inhibition of Phosphodiesterase-2 in Mice. J. Pharmacol. Exp. Ther. 326, 369–379. 10.1124/jpet.108.137208 18456873 PMC2913601

[B55] Mende M. Fletcher E. V. Belluardo J. L. Pierce J. P. Bommareddy P. K. Weinrich J. A. (2016). Sensory-Derived Glutamate Regulates Presynaptic Inhibitory Terminals in Mouse Spinal Cord. Neuron 90, 1189–1202. 10.1016/j.neuron.2016.05.008 27263971 PMC4912012

[B56] Mezuk B. Eaton W. W. Albrecht S. Golden S. H. (2008). Depression and Type 2 Diabetes over the Lifespan: a Meta-Analysis. Diabetes Care 31, 2383–2390. 10.2337/dc08-0985 19033418 PMC2584200

[B57] Möhler H. (2012). The GABA System in Anxiety and Depression and its Therapeutic Potential. Neuropharmacology 62, 42–53. 10.1016/j.neuropharm.2011.08.040 21889518

[B58] Moreau J. L. (2002). Simulating the Anhedonia Symptom of Depression in Animals. Dialogues Clin. Neurosci. 4, 351–360. 10.31887/DCNS.2002.4.4/jlmoreau 22034464 PMC3181703

[B59] Morishita S. (2009). Clonazepam as a Therapeutic Adjunct to Improve the Management of Depression: a Brief Review. Hum. Psychopharmacol. 24, 191–198. 10.1002/hup.1015 19330803

[B60] Murrough J. W. Abdallah C. G. Mathew S. J. (2017). Targeting Glutamate Signalling in Depression: Progress and Prospects. Nat. Rev. Drug Discov. 16, 472–486. 10.1038/nrd.2017.16 28303025

[B61] Papakostas G. I. Shelton R. C. (2008). Use of Atypical Antipsychotics for Treatment-Resistant Major Depressive Disorder. Curr. Psychiatry Rep. 10, 481–486. 10.1007/s11920-008-0077-3 18980731

[B62] Peng G. J. Tian J. S. Gao X. X. Zhou Y. Z. Qin X. M. (2015). Research on the Pathological Mechanism and Drug Treatment Mechanism of Depression. Curr. Neuropharmacol 13, 514–523. 10.2174/1570159x1304150831120428 26412071 PMC4790409

[B63] Perez-Riverol Y. Csordas A. Bai J. Bernal-Llinares M. Hewapathirana S. Kundu D. J. (2019). The PRIDE Database and Related Tools and Resources in 2019: Improving Support for Quantification Data. Nucleic Acids Res. 47, D442–d450. 10.1093/nar/gky1106 30395289 PMC6323896

[B64] Porcu A. Vaughan M. Nilsson A. Arimoto N. Lamia K. Welsh D. K. (2020). Vulnerability to Helpless Behavior Is Regulated by the Circadian Clock Component CRYPTOCHROME in the Mouse Nucleus Accumbens. Proc. Natl. Acad. Sci. U S A. 117, 13771–13782. 10.1073/pnas.2000258117 32487727 PMC7306774

[B65] Qiu J. Hu S. Y. Shi G. Q. Wang S. E. (2014a). Changes in Regional Cerebral Blood Flow with Chaihu-Shugan-San in the Treatment of Major Depression. Pharmacogn Mag. 10, 503–508. 10.4103/0973-1296.141775 25422553 PMC4239730

[B66] Qiu J. Hu S. Y. Zhang C. H. Shi G. Q. Wang S. E. Xiang T. (2014b). The Effect of Chaihu-Shugan-San and its Components on the Expression of ERK5 in the hippocampus of Depressed Rats. J. Ethnopharmacol 152, 320–326. 10.1016/j.jep.2014.01.004 24486208

[B67] Ramakrishnan N. A. Drescher M. J. Drescher D. G. (2012). The SNARE Complex in Neuronal and Sensory Cells. Mol. Cel Neurosci 50, 58–69. 10.1016/j.mcn.2012.03.009 PMC3570063 22498053

[B68] Reierson G. W. Guo S. Mastronardi C. Licinio J. Wong M. L. (2011). cGMP Signaling, Phosphodiesterases and Major Depressive Disorder. Curr. Neuropharmacol 9, 715–727. 10.2174/157015911798376271 22654729 PMC3263465

[B69] Ren Y. Hao P. Dutta B. Cheow E. S. Sim K. H. Gan C. S. (2013). Hypoxia Modulates A431 Cellular Pathways Association to Tumor Radioresistance and Enhanced Migration Revealed by Comprehensive Proteomic and Functional Studies. Mol. Cel Proteomics 12, 485–498. 10.1074/mcp.M112.018325 PMC3567868 23204318

[B70] Scheid V. (2013). Depression, Constraint, and the Liver: (Dis)assembling the Treatment of Emotion-Related Disorders in Chinese Medicine. Cult. Med. Psychiatry 37, 30–58. 10.1007/s11013-012-9290-y 23315392 PMC3586067

[B71] Snyder J. S. Soumier A. Brewer M. Pickel J. Cameron H. A. (2011). Adult Hippocampal Neurogenesis Buffers Stress Responses and Depressive Behaviour. Nature 476, 458–461. 10.1038/nature10287 21814201 PMC3162077

[B72] Sonnewald U. Olstad E. Qu H. Babot Z. Cristòfol R. Suñol C. (2004). First Direct Demonstration of Extensive GABA Synthesis in Mouse Cerebellar Neuronal Cultures. J. Neurochem. 91, 796–803. 10.1111/j.1471-4159.2004.02794.x 15525333

[B73] Stratton B. S. Warner J. M. Wu Z. Nikolaus J. Wei G. Wagnon E. (2016). Cholesterol Increases the Openness of SNARE-Mediated Flickering Fusion Pores. Biophys. J. 110, 1538–1550. 10.1016/j.bpj.2016.02.019 27074679 PMC4833774

[B74] Wang C. Tu J. Zhang S. Cai B. Liu Z. Hou S. (2020). Different Regions of Synaptic Vesicle Membrane Regulate VAMP2 Conformation for the SNARE Assembly. Nat. Commun. 11, 1531. 10.1038/s41467-020-15270-4 32210233 PMC7093461

[B75] Wang Y. Fan R. Huang X. (2012). Meta-analysis of the Clinical Effectiveness of Traditional Chinese Medicine Formula Chaihu-Shugan-San in Depression. J. Ethnopharmacol 141, 571–577. 10.1016/j.jep.2011.08.079 21933701

[B76] Wang Y. Li M. Liang Y. Yang Y. Liu Z. Yao K. (2017). Chinese Herbal Medicine for the Treatment of Depression: Applications, Efficacies and Mechanisms. Curr. Pharm. Des. 23, 5180–5190. 10.2174/1381612823666170918120018 28925891

[B77] Wang Y. S. Shen C. Y. Jiang J. G. (2019). Antidepressant Active Ingredients from Herbs and Nutraceuticals Used in TCM: Pharmacological Mechanisms and Prospects for Drug Discovery. Pharmacol. Res. 150, 104520. 10.1016/j.phrs.2019.104520 31706012

[B78] Wilhelm M. Schlegl J. Hahne H. Gholami A. M. Lieberenz M. Savitski M. M. (2014). Mass-spectrometry-based Draft of the Human Proteome. Nature 509, 582–587. 10.1038/nature13319 24870543

[B79] Xing H. Zhang K. Zhang R. Shi H. Bi K. Chen X. (2015). Antidepressant-like Effect of the Water Extract of the Fixed Combination of Gardenia Jasminoides, Citrus Aurantium and Magnolia Officinalis in a Rat Model of Chronic Unpredictable Mild Stress. Phytomedicine 22, 1178–1185. 10.1016/j.phymed.2015.09.004 26598917

[B80] Xing H. Zhang X. Xing N. Qu H. Zhang K. (2019). Uncovering Pharmacological Mechanisms of Zhi-Zi-Hou-Po Decoction in Chronic Unpredictable Mild Stress Induced Rats through Pharmacokinetics, Monoamine Neurotransmitter and Neurogenesis. J. Ethnopharmacol 243, 112079. 10.1016/j.jep.2019.112079 31302206

[B81] Xu Y. Pan J. Chen L. Zhang C. Sun J. Li J. (2013). Phosphodiesterase-2 Inhibitor Reverses Corticosterone-Induced Neurotoxicity and Related Behavioural Changes via cGMP/PKG Dependent Pathway. Int. J. Neuropsychopharmacol. 16, 835–847. 10.1017/s146114571200065x 22850435

[B82] Yan L. Xu X. He Z. Wang S. Zhao L. Qiu J. (2020). Antidepressant-Like Effects and Cognitive Enhancement of Coadministration of Chaihu Shugan San and Fluoxetine: Dependent on the BDNF-ERK-CREB Signaling Pathway in the Hippocampus and Frontal Cortex. Biomed. Res. Int. 2020, 2794263. 10.1155/2020/2794263 32185198 PMC7060874

[B83] Yang Y. Hu Z. Du X. Davies H. Huo X. Fang M. (2017). miR-16 and Fluoxetine Both Reverse Autophagic and Apoptotic Change in Chronic Unpredictable Mild Stress Model Rats. Front. Neurosci. 11, 428. 10.3389/fnins.2017.00428 28790887 PMC5524920

[B84] Yankelevitch-Yahav R. Franko M. Huly A. Doron R. (2015). The Forced Swim Test as a Model of Depressive-like Behavior. J. Visual. Exp. JoVE 97. 10.3791/52587 PMC4401172 25867960

[B85] Yeung W. F. Chung K. F. Ng K. Y. Yu Y. M. Ziea E. T. Ng B. F. (2014). A Systematic Review on the Efficacy, Safety and Types of Chinese Herbal Medicine for Depression. J. Psychiatr. Res. 57, 165–175. 10.1016/j.jpsychires.2014.05.016 24974002

[B86] Zhang J. Yang M. K. Zeng H. Ge F. (2016). GAPP: A Proteogenomic Software for Genome Annotation and Global Profiling of Post-translational Modifications in Prokaryotes. Mol. Cel Proteomics 15, 3529–3539. 10.1074/mcp.M116.060046 PMC5098048 27630248

[B87] Zhang J. H. Zhu Y. Fan X. H. Zhang B. L. (2015). Efficacy-oriented Compatibility for Component-Based Chinese Medicine. Acta Pharmacol. Sin 36, 654–658. 10.1038/aps.2015.8 25864650 PMC4594187

[B88] Zhang L. Yu Z. Wang Y. Wang X. Zhang L. Wang C. (2016). Quantitative Proteomics Reveals Molecular Mechanism of Gamabufotalin and its Potential Inhibition on Hsp90 in Lung Cancer. Oncotarget 7, 76551–76564. 10.18632/oncotarget.10388 27384878 PMC5363529

[B89] Zhang Y. Yuan S. Pu J. Yang L. Zhou X. Liu L. (2018). Integrated Metabolomics and Proteomics Analysis of Hippocampus in a Rat Model of Depression. Neuroscience 371, 207–220. 10.1016/j.neuroscience.2017.12.001 29237567

[B90] Zhang Y. J. Huang X. Wang Y. Xie Y. Qiu X. J. Ren P. (2011). Ferulic Acid-Induced Anti-depression and Prokinetics Similar to Chaihu-Shugan-San via Polypharmacology. Brain Res. Bull. 86, 222–228. 10.1016/j.brainresbull.2011.07.002 21791239

[B91] Zhou D. Liu J. Hang Y. Li T. Li P. Guo S. (2020). TMT-based Proteomics Analysis Reveals the Protective Effects of Xuefu Zhuyu Decoction in a Rat Model of Traumatic Brain Injury. J. Ethnopharmacol 258, 112826. 10.1016/j.jep.2020.112826 32298754

[B92] Zhu X. Li W. Li Y. Xu W. Yuan Y. Zheng V. (2019). The Antidepressant- and Anxiolytic-like Effects of Resveratrol: Involvement of phosphodiesterase-4D Inhibition. Neuropharmacology 153, 20–31. 10.1016/j.neuropharm.2019.04.022 31026437

