# Supplementary material for: Proteomics Study Reveals the Anti-Depressive Mechanisms and the Compatibility Advantage of Chaihu-Shugan-San in a Rat Model of Chronic Unpredictable Mild Stress
Source: Front Pharmacol. 2022 Jan 17;12:791097. doi: 10.3389/fphar.2021.791097 (PMC8802092; doi:10.3389/fphar.2021.791097)
Supplement: Supplementary file 1 [file DataSheet1.ZIP › Supplementary Material.docx]

Supplementary Material


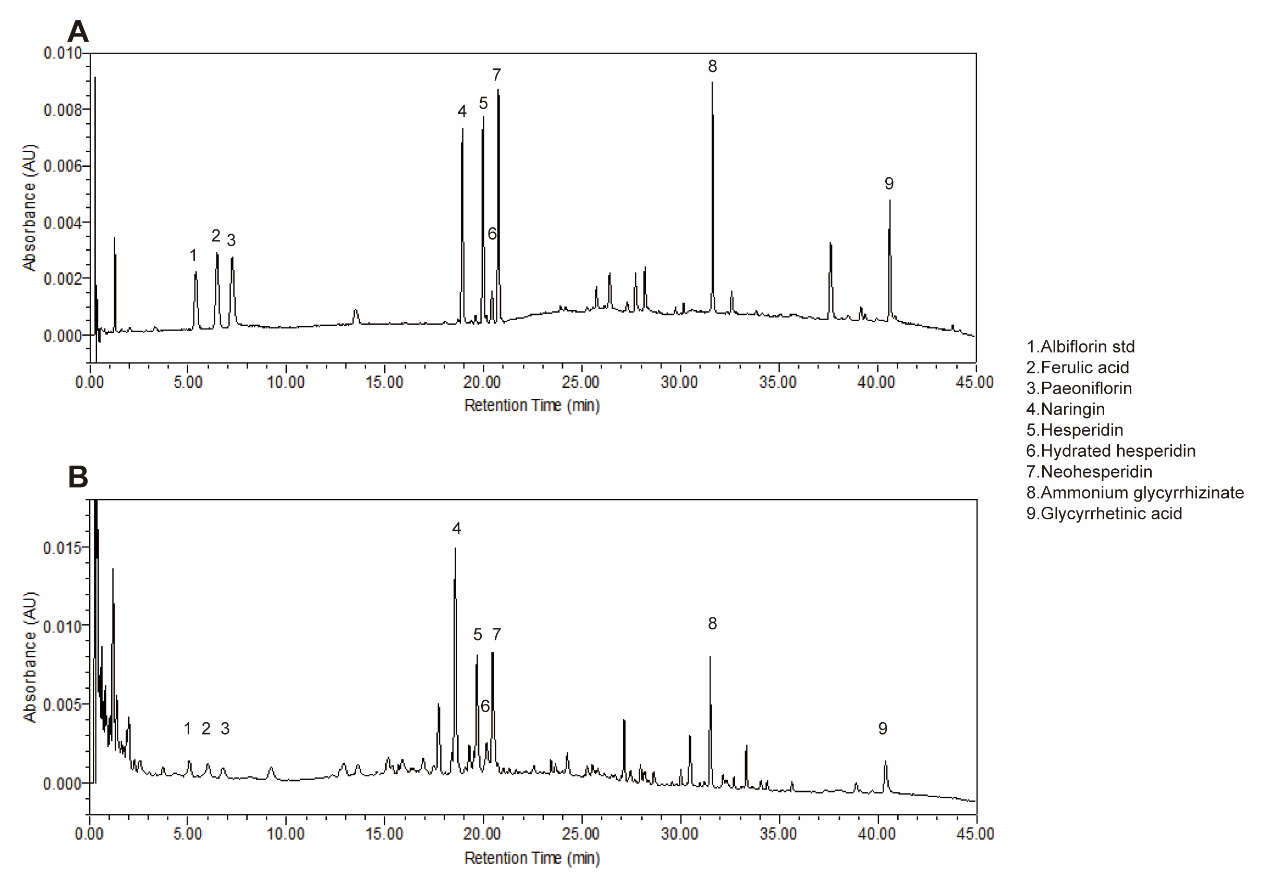


**Figure S1.** Ultra-performance liquid chromatography (UPLC) analysis of Chaihu-Shugan-San components. A: the chromatogram of 9 reference standards B: sample chromatogram. (1. Albiflorin std; 2. Ferulic acid; 3. Paeoniflorin; 4. Naringin; 5. Hesperidin; 6. Hydrated hesperidin; 7. Neohesperidin; 8. Ammonium glycyrrhizinate; 9. Glycyrrhetinic acid)


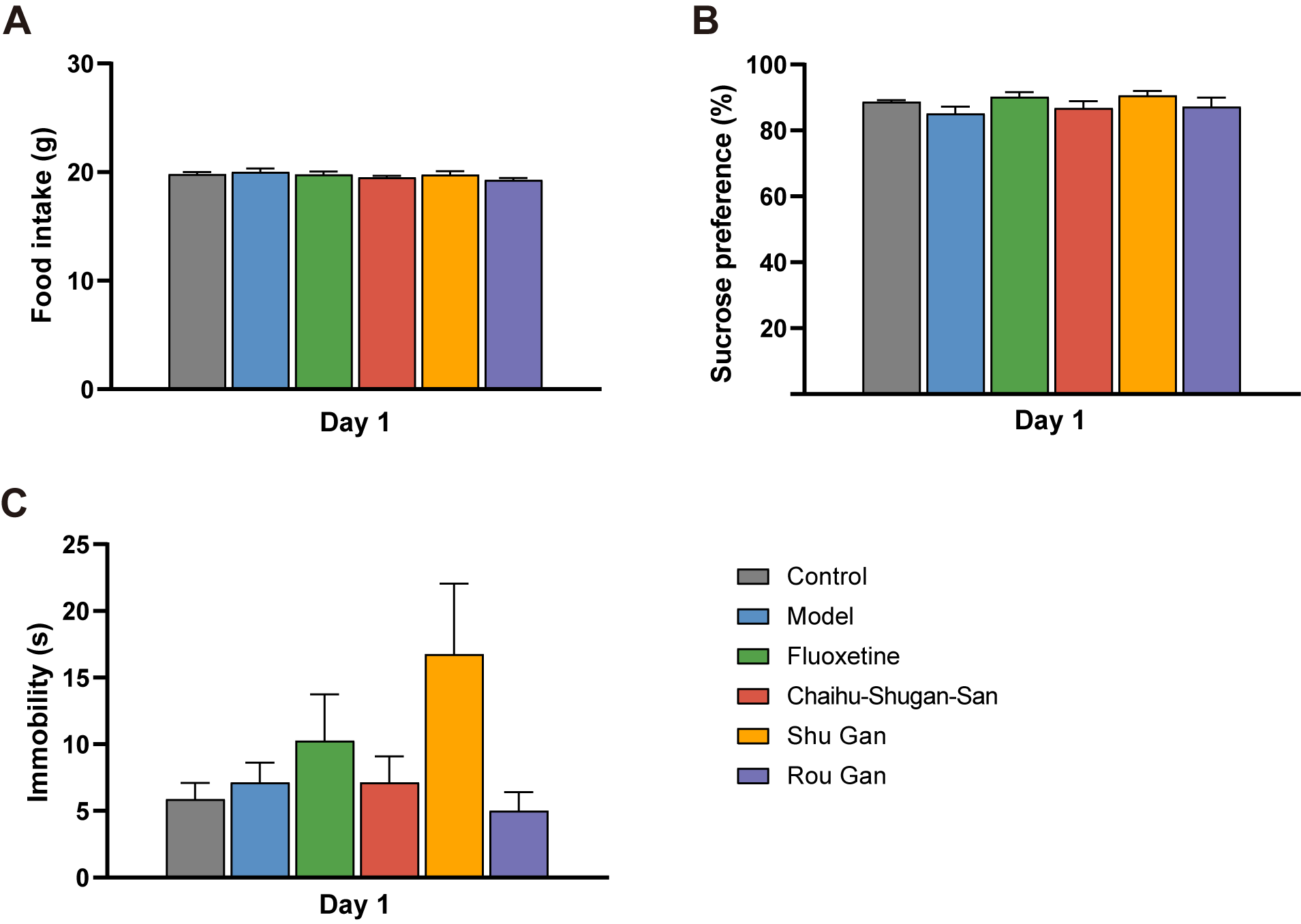


**Figure S2.** Food intake (A), sucrose consumption tests (B) and forced swim test (C) were performed on day 1 (n=8, ∗*p*<0.05, ∗∗*p*<0.01). All data are expressed as mean±SEM.


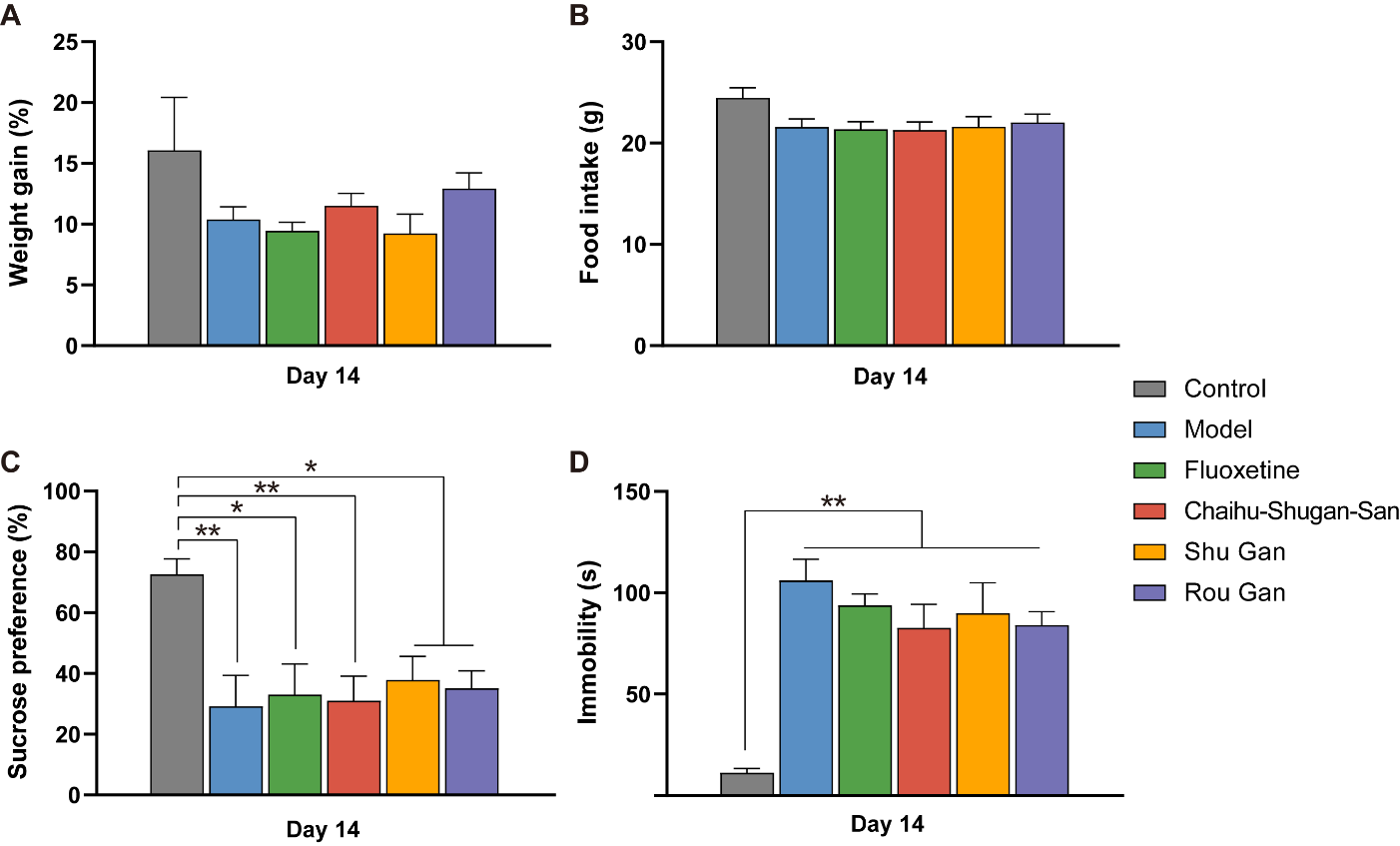


**Figure S3.** Bodyweight gain (A), food intake (B), sucrose consumption tests (C) and forced swim test (D) were performed on day 14 (n=8, ∗*p*<0.05, ∗∗*p*<0.01). All data are expressed as mean±SEM.


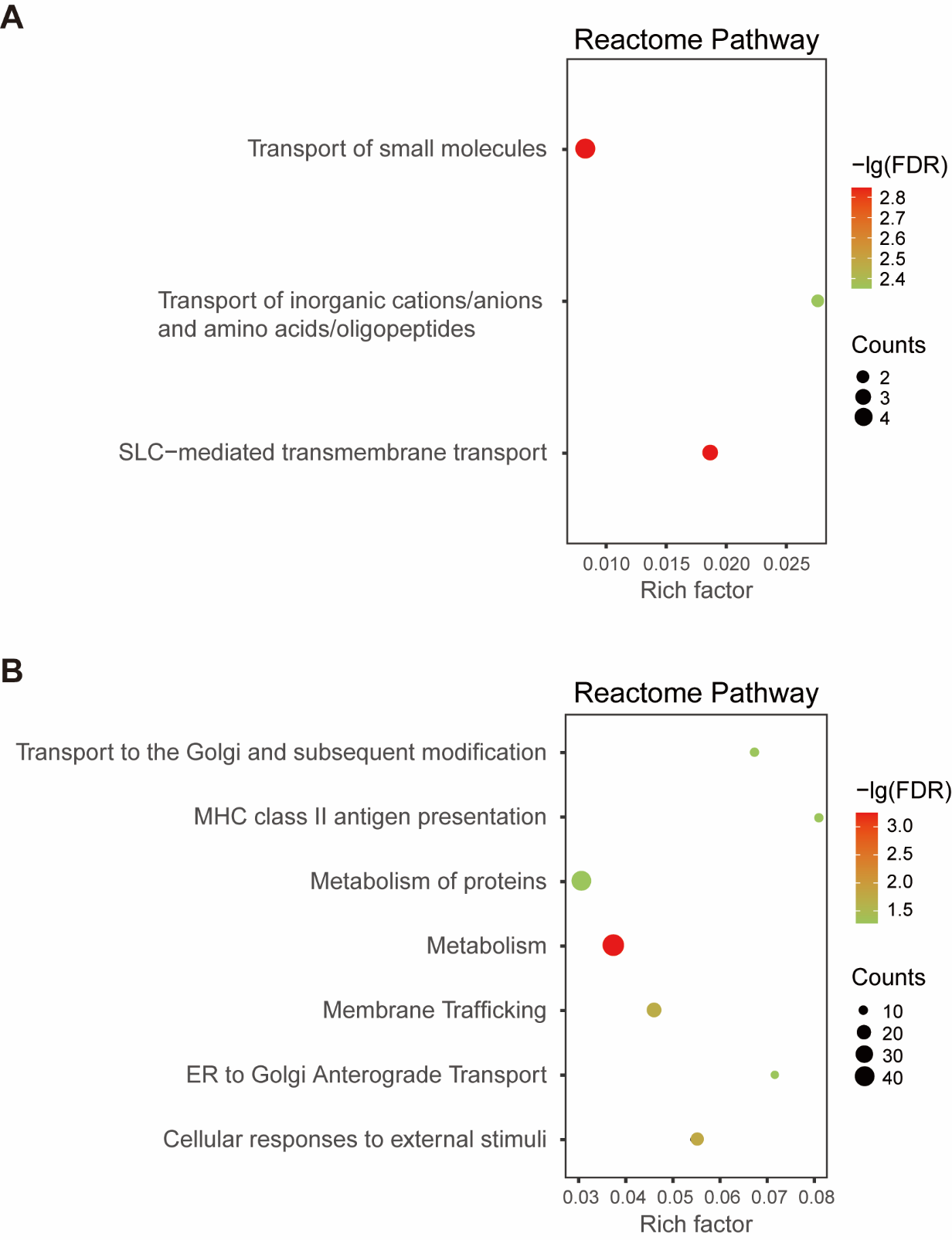


**Figure S4.** (A) Reactome pathways analysis of 12 DEPs in Shu Gan. (B) Reactome pathways analysis of 407 DEPs in Rou Gan.
